# Demystifying the Neuroprotective Role of Neuropeptides in Parkinson’s Disease: A Newfangled and Eloquent Therapeutic Perspective

**DOI:** 10.3390/ijms23094565

**Published:** 2022-04-20

**Authors:** Tapan Behl, Piyush Madaan, Aayush Sehgal, Sukhbir Singh, Hafiz A. Makeen, Mohammed Albratty, Hassan A. Alhazmi, Abdulkarim M. Meraya, Simona Bungau

**Affiliations:** 1 Chitkara College of Pharmacy, Chitkara University, Rajpura 140401, India; piyushmadaan4811@gmail.com (P.M.); aayushsehgal00@gmail.com (A.S.); sukhbir.singh@chitkara.edu.in (S.S.); 2 Pharmacy Practice Research Unit, Department of Clinical Pharmacy, College of Pharmacy, Jazan University, Jazan 45142, Saudi Arabia; hafiz@jazanu.edu.sa (H.A.M.); ameraya@jazanu.edu.sa (A.M.M.); 3 Department of Pharmaceutical Chemistry, College of Pharmacy, Jazan University, Jazan 45142, Saudi Arabia; malbratty@jazan.edu.sa (M.A.); hasalhazmi@gmail.com (H.A.A.); 4 Substance Abuse and Toxicology Research Center, Jazan University, Jazan 45142, Saudi Arabia; 5 Department of Pharmacy, Faculty of Medicine and Pharmacy, University of Oradea, 410028 Oradea, Romania; 6 Doctoral School of Biomedical Sciences, University of Oradea, 410028 Oradea, Romania

**Keywords:** Parkinson’s disease, neuropeptides, substance P, ghrelin, neuropeptide Y, neurotensin, pituitary adenylate cyclase-activating polypeptide, neuroprotective action

## Abstract

Parkinson’s disease (PD) refers to one of the eminently grievous, preponderant, tortuous nerve-cell-devastating ailments that markedly impacts the dopaminergic (DArgic) nerve cells of the midbrain region, namely the substantia nigra pars compacta (SN-PC). Even though the exact etiopathology of the ailment is yet indefinite, the existing corroborations have suggested that aging, genetic predisposition, and environmental toxins tremendously influence the PD advancement. Additionally, pathophysiological mechanisms entailed in PD advancement encompass the clumping of α-synuclein inside the lewy bodies (LBs) and lewy neurites, oxidative stress, apoptosis, neuronal-inflammation, and abnormalities in the operation of mitochondria, autophagy lysosomal pathway (ALP), and ubiquitin–proteasome system (UPS). The ongoing therapeutic approaches can merely mitigate the PD-associated manifestations, but until now, no therapeutic candidate has been depicted to fully arrest the disease advancement. Neuropeptides (NPs) are little, protein-comprehending additional messenger substances that are typically produced and liberated by nerve cells within the entire nervous system. Numerous NPs, for instance, substance P (SP), ghrelin, neuropeptide Y (NPY), neurotensin, pituitary adenylate cyclase-activating polypeptide (PACAP), nesfatin-1, and somatostatin, have been displayed to exhibit consequential neuroprotection in both in vivo and in vitro PD models via suppressing apoptosis, cytotoxicity, oxidative stress, inflammation, autophagy, neuronal toxicity, microglia stimulation, attenuating disease-associated manifestations, and stimulating chondriosomal bioenergetics. The current scrutiny is an effort to illuminate the neuroprotective action of NPs in various PD-experiencing models. The authors carried out a methodical inspection of the published work procured through reputable online portals like PubMed, MEDLINE, EMBASE, and Frontier, by employing specific keywords in the subject of our article. Additionally, the manuscript concentrates on representing the pathways concerned in bringing neuroprotective action of NPs in PD. In sum, NPs exert substantial neuroprotection through regulating paramount pathways indulged in PD advancement, and consequently, might be a newfangled and eloquent perspective in PD therapy.

## 1. Introduction

Parkinson’s disease (PD), a clinical state portrayed around 20.5 decades ago by an English surgeon named James Parkinson as paralysis agitans, is currently acknowledged as the second leading, mystifying, and incapacitating neurodegenerative disease in older individuals [1]. The condition is represented by a tetrad of cardinal manifestations, viz., rigor, tremor, postural deformities, and bradykinesia [2,3]. The aforenamed pivotal manifestations are ascribable to the deterioration of dopaminergic (DArgic) nerve cells in the substantia nigra pars compacta (SN-PC), a region pinpointed in the midbrain [4]. With a tremendous upsurge in the prevalence and incidence rates across the different regions of the nation, PD is emerging as a grievous age-associated and intricate malady [5,6]. Owing to the profound nerve cell protection exhibited by estrogen in females, the females display a de-escalated possibility of encountering PD comparably to males [7]. Although the exact etiopathogenesis of the malady remains perplexing, multifaceted, and vague, extensive data robustly propounds that aging, genetic predisposition, and subjection to environmental toxins unitedly partake in the progression of the malady [8,9,10,11,12,13,14]. The pathophysiological processes embroiled in PD comprehends the clumping of α-synuclein inside the lewy bodies (LBs) and lewy neurites, oxidative stress, apoptosis, neuronal-inflammation, and abnormalities in the operation of mitochondria, autophagy lysosomal pathway (ALP), and ubiquitin–proteasome system (UPS). However, the interrelationship among these processes is still inexplicit [8,15]. Up to the present time, the treatment with the assistance of dopamine (DA) precursor (levodopa), DA agonists, catechol-O-methyltransferase (COMT) inhibitors, and monoamine oxidase B (MAO-B) inhibitors principally focuses on the mitigation of PD-related manifestations, but hitherto no therapeutic candidate has been indicated to totally abolish the progression of the ailment [16,17,18].

Neuropeptides (NPs) are designated as tiny, protein-comprising additional messenger substances that are fundamentally generated and liberated by nerve cells inside the entire nervous system, comprehending the central nervous system (CNS) and the peripheral nervous system (PNS) [19,20]. NPs are synthesized in the cell body from their large protein precursors denominated as prepropeptides (which are synthesized on palade granules at the endoplasmic reticulum and processed by means of Golgi complex). The NPs are principally transcripted and translated from the prepropeptides genes. Further, the activation of prepropeptides carried by proteases/proteinases (peptide bonds hydrolyzing biocatalysts) culminates in the conversion of prepropeptides into propeptides, and at last, following the stimulation of converting biocatalysts, NPs are procured [21,22,23]. Proteolytic processing has been reported to significantly partake in the activation, partial inactivation, or inactivation of the modulatory peptides, for instance, NPs. Proteases, otherwise denominated as proteinases, carry out the breakdown, and as a consequence, might activate, inactivate, or liberate other proteins/peptides [24,25]. These modulatory proteases are fundamentally pinpointed on the surface of the cell or are liberated by the cells. Although the duo forms of proteases tend to perform identical operations, the existing literature has elucidated that cell-surface proteases possess significantly greater regulatory and specialized operations in comparison to those proteases which are liberated by the cells [25]. The cell-surface proteases exert their action by carrying out deterioration of the duo, i.e., the bioactive peptides, and the cellular operations. Apart from the proteolytic deterioration, and conjugation and oxidation reactions, the eradication via filtration/diffusion, is of paramount importance [25]. The majority of the cell-surface proteases have been originally marked as the clearing biocatalysts despite the fact that they possess the aptitude to break the peptides having not more than eighty residues. Currently, they are considered as the regulatory proteases (for instance, angiotensin-converting enzyme (ACE), endothelin-converting enzyme (ECE), neutral endopeptidase (NEP), and dipeptidyl peptidase IV (DPP IV)) that hold the enormous aptitude to modulate the activation/inactivation of NPs [23]. Following their synthesis, NPs are stored/packaged in the large and dense vesicles, and finally their liberation is facilitated by means of an expulsion process termed exocytosis (following depolarization of the cell) [21,22,23]. Thereafter, NPs undergo interaction with receptors, namely G-protein coupled receptors (GPCRs), in order to instigate their physiological actions and regulate nerve cell operation [19,26]. The NPs and their seven transmembrane domain (7TM) receptors/GPCRs are pinpointed ubiquitously in the body, and they usually exist in amalgamation with classic neurotransmitters [18,20,21,27,28]. NPs consequentially partake in the modulation of the immune system, biological equilibrium (for instance, biotransformation of blood sugar, blood pressure, equilibrium in the water content, feeding behavior, stress reaction, and pain), and neuronal protection [29]. Presently, numerous NPs have been elucidated to exhibit substantial neuronal protection in both in vivo and in vitro models of PD, for instance, substance P (SP) [30], ghrelin [31,32], neuropeptide Y (NPY) [33], neurotensin [34], pituitary adenylate cyclase-activating polypeptide (PACAP) [35], nesfatin-1 [36], and somatostatin (SST) [37] via suppressing apoptosis, cytotoxicity, oxidative stress, autophagy, inflammation, nerve cell toxicity, microglia stimulation, attenuating disease-associated manifestations, and stimulating chondriosomal bioenergetics.

In the current scrutiny, the authors attempt to enlighten the linkage between the aforenamed NPs and PD, and elucidate the pathways by means of which these NPs contribute to significant nerve cell protection in PD. A comprehensive examination has been carried out by means of existing literature, i.e., both review and research articles, which were searched through esteemed and well-renowned medical databases, for instance, PubMed, MEDLINE, EMBASE, Frontier, etc., by employing particular keywords in the theme of our paper. The outcome is an explanatory work that would be a tremendously valuable resource for upcoming papers in this respective discipline.

## 2. Understanding the Etiopathogenic Pathways Underlying Parkinson’s Disease

**Figure 1:**
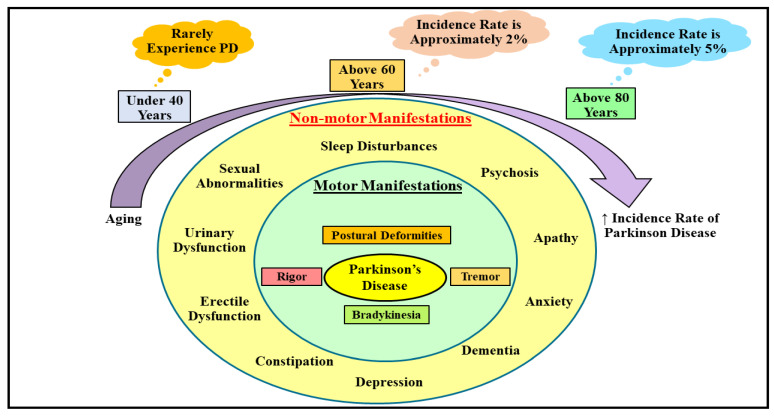
The diagram illustrates the expansion in the incidence rate of Parkinson’s disease with aging, and also outlines the motor and nonmotor manifestations associated with Parkinson’s disease. Aging is reckoned as the critical parameter actively engaged in the evolution of PD. The incidence rate of PD escalates with aging, i.e., people ranging under 30–40 years rarely experience PD, 2% of people ranging above 60–70 years experience PD, and 5% of people ranging above 80–90 years experience PD. The motor manifestations of the disease comprehends rigor, postural deformities, tremor, and bradykinesia. On the other hand, the nonmotor manifestations associated with the disease comprehends urinary dysfunction, sexual abnormalities, sleep disturbances, psychosis, dementia, anxiety, apathy, depression, constipation, and erectile dysfunction. Howbeit, the nonmotor manifestations are comparably less noticeable than motor manifestations. PD, Parkinson’s disease; ↑, increasing.

PD, a mystified, multifaceted, and debilitating malady, is depicted by the devastation of DArgic nerve cells inside the SN-PC (pinpointed in the midbrain region), which eventually contributes to DA scantiness in the striatal region. It has been elucidated that deposition of a protein termed α-synuclein within the LBs and lewy neurites is thought to be the characteristic neuropathogenic hallmark of PD [38]. Generally, PD is marked as a motor system ailment exhibiting a quadriad of imperative manifestations, comprehending rigor (stiffness), tremor (shaking in the hands, feets, and legs), postural deformities (abnormal balance and body posture), and bradykinesia (slowed/difficult movement). Nonmotor manifestations, such as urinary and sexual abnormalities, sleep disturbances, psychosis, dementia, anxiety, apathy, depression, constipation, and erectile dysfunction, are also encountered by individuals suffering from PD; however, they are somewhat less apparent in comparison to motor manifestations (Figure 1) [39].

### 2.1. Understanding the Etiological Processes Underlying Parkinson’s Disease

Analogous to other frequently emerging age-associated nerve cell deteriorating ailments, PD also commences owing to the amalgamation of the trio, namely aging, genetic predisposition, and subjection to environmental toxins, and is recklessly impacting the global economy [40,41]. According to a meta-analysis, the incidence of PD is expanding at an alarming rate in advanced and well-established parts of the nation, and it has been revealed to escalate abruptly with aging [42]. Another investigation has demonstrated that people falling under the age range of 30–40 years rarely experience PD, while it hits nearly 2% of people going above 60–70 years of age grade, and nearly 5% of people falling above the age range of 80–90 years nationwide (Figure 1) [43]. Even the gender differences enormously impact the PD emergence, i.e., males are comparably more prone to PD than females because of the nerve cell safeguarding action of estrogen in females in the initial stages of the disease [44,45]. There are no further gender differences in PD following its progression to the disastrous or later/severe stages, although estrogen partakes in bringing considerable neuronal protection in PD, and it renders no safeguarding effects following the commencement of clinical manifestations [45].

**Figure 2:**
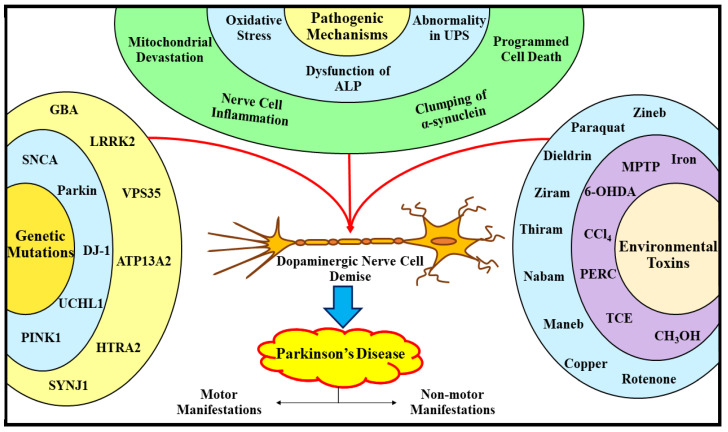
Portraying the active participation of genetic mutations, environmental toxins, and pathogenic processes in the progression/evolvement of Parkinson’s disease. The trio, namely genetic mutations (*SNCA*, *Parkin*, DJ-1, *UCHL1*, *PINK1*, *GBA*, *LRRK2*, VPS35, ATP13A2, HTRA2, and SYNJ1), subjection to environmental toxins (MPTP, 6-OHDA, CH_3_OH, PERC, TCE, CCl_4_, zineb, paraquat, rotenone, dieldrin, ziram, thiram, nabam, maneb, copper, and iron), and pathogenic mechanisms (oxidative stress, dysfunction of ALP, abnormality in UPS, mitochondrial devastation, nerve cell inflammation, clumping of α-synuclein, and programmed cell death), give rise to dopaminergic nerve cell demise, which as a result culminates in the progression/evolvement of PD. *SNCA*, α-synuclein; *Parkin*, *Parkin RBR E3 ubiquitin–protein ligase*; DJ-1, protein deglycase; *UCHL1*, *ubiquitin carboxy* (C)*-terminal hydrolase L1*; *PINK1*, *PTEN-induced kinase 1*; *GBA*, glucocerebrosidase; *LRRK2*, *leucine-rich repeat kinase 2*; VPS35, vacuolar protein sorting 35; ATP13A2, neuronal P-type adenosine triphosphate (ATP)ase gene; HTRA2, high temperature requirement A2; SYNJ1, *synaptojanin 1*; MPTP, 1-methyl-4-phenyl-1,2,3,6-tetrahydropyridine; 6-OHDA, 6-hydroxy DA; CH_3_OH, methanol; PERC, perchloroethylene; TCE, trichloroethylene; CCl_4_, carbon tetrachloride; ALP, autophagy lysosomal pathway; UPS, ubiquitin-proteasome system; PD, Parkinson’s disease.

Apart from aging, the duo, namely genetic profile and subjection to environmental toxins, are recognized as fundamental participants indulged in the commencement and advancement of different forms of PD, by triggering DArgic nerve cell demise, as illustrated in Figure 2. Over the last few years, a profusion of exploration in the domain of PD has culminated in the elucidation that nearly 5–10% of the delayed commencement sorts of the PD are immensely related with genetic mutations [46]. To date, numerous genes have been reported to be implicated in the PD evolution, encompassing α-synuclein (*SNCA*) [46,47,48] *Parkin RBR E3 ubiquitin–protein ligase* (*Parkin*) [49], *ubiquitin carboxy* (C)*-terminal hydrolase L1* (*UCHL1*) [50], *PTEN-induced kinase 1* (*PINK1*) [51], protein deglycase (DJ-1) [52], *leucine-rich repeat kinase 2* (*LRRK2*) [53], glucocerebrosidase (*GBA*) [54], vacuolar protein sorting 35 (VPS35) [55], neuronal P-type adenosine triphosphate (ATP)ase gene (ATP13A2) [56], high temperature requirement A2 (HTRA2) [57], and synaptojanin 1 (SYNJ1) [58]. In addition, a plethora of corroborations profoundly indicate that exposure to nerve cell toxic agents (1-methyl-4-phenyl-1,2,3,6-tetrahydropyridine (MPTP) [38,51,52], and 6-hydroxy DA (6-OHDA) [38,51]), pesticides (paraquat) [46,59,60], rotenone [46,59,60], dieldrin [61], zineb [46,62], ziram [46,63] and thiram [64], fungicides (nabam [65], and maneb [65]), and solvents (methanol (CH_3_OH) [66], perchloroethylene (PERC) [66,67], trichloroethylene (TCE) [66,67], and carbon tetrachloride (CCl_4_) [66,67], considerably escalate the expansion of PD (via triggering the demise of DArgic nerve cells), and the concomitant range of motor as well as nonmotor manifestations. In addition, a recent study has revealed that transition metals, in particular iron (Fe) and copper (Cu), via elevating the oxidative stress, lipid peroxidation, and clumping of α-synuclein inside the LBs, substantially contribute to the progression of PD [68].

### 2.2. Understanding the Pathogenic Processes Underlying Parkinson’s Disease

Even though the pathogenesis underlying PD remains mystified and equivocal, a myriad of scrutinizations in the foregone years led to the unfolding of several pathways of paramount importance that markedly participate in the PD progression. These comprehend oxidative stress, dysfunction of the ALP, abnormality in the UPS, mitochondrial devastation, nerve cell inflammation, clumping of α-synuclein, and programmed cell death/apoptosis (portrayed in Figure 2) [8,15,41,46,59,68].

#### 2.2.1. Oxidative Stress and Parkinson’s Disease

Mounting corroborations elucidated that, amidst the multiple processes deeply entangled in the pathogenesis of PD, oxidative stress has reaped a noteworthy prominence. Pursuant to one of the widely acknowledged theories, namely the free radical theory, or otherwise denominated as the oxidative stress theory (which was propounded by a renowned biochemist named Denham Harman during the mid-20th century), the chondriosome/power plants of the cell (mitochondria) are reckoned to be a “hotspot” for degenerative events [69]. The investigators set forth that an anomalous complex-I operation inside the chondriosome has been detected in the case of PD, that significantly intercedes with the ATP formation within the cells, and in turn culminates in cellular demise [70]. Further, the nitrogen-comprising low molar mass compounds denominated as biogenic amines (BAs) pinpointed inside the brain, for instance, DA and 5-hydroxytryptamine (5-HT)/serotonin, have been expounded to exhibit consequential antioxidant/free-radical scavenging abilities [71]. Howbeit, the DA fragmentation precipitated by MAO-B, in amalgamation with oxygen (O_2_) existent in the stable ground state, markedly contributes to the generation of eminently reactive, pernicious, and unstable substances denominated as oxygen radicals/reactive oxygen species (ROS) [72]. Furthermore, an inspection of human brain autopsies has displayed a consequential plummet in the quantities of an imperative tripeptide antioxidant designated as glutathione (GSH), an upsurge in the Fe and malondialdehyde (MDA) quantities, and oxidative harm to macromolecules (lipids, and polypeptides) [73,74,75]. Another study has spotted that those individuals with PD exhibit plummeted functioning of a biocatalyst with antioxidant abilities, termed catalase (CAT), and an upsurge in the lipid hydroperoxides (LOOH), MDA, and the functioning of a biocatalyst possessing antioxidant abilities, designated as superoxide dismutase (SOD) [76]. In accordance with these examinations, MDA is speculated to be the significant biomarker of the malady, while SOD and LOOH are profoundly related to delayed manifestations of the malady. Moreover, several investigations on human beings and animal models with PD have elucidated that a signaling molecule, namely nitrogen monoxide/nitric oxide (NO), significantly participates in multiple pathogenic mechanisms, viz., inflammatory processes, oxidative damage, deoxyribonucleic acid (DNA) devastation, excitotoxicity, S-nitrosylation of numerous proteins, and mitochondrial impairment, and eventually culminates into nerve cell deterioration [76,77,78]. The aforestated explorations markedly highlight the participation of oxidative stress in the PD progression.

#### 2.2.2. Autophagy Lysosomal Pathway Dysfunction and Parkinson’s Disease

Existing data has promulgated that, in the case of PD, numerous ALP-related components have been found to be considerably plummeted or undermined, which displays an immense resemblance to the UPS pathway results. Published literature has elucidated that fundamental and eminent protein constituent of the single phospholipid bilayer (lysosomal membrane), encompassing lysosome-associated membrane protein 1 (LAMP1) and lysosome-associated membrane protein 2A (LAMP2A), and heat shock proteins (HSPs), otherwise denominated as molecular chaperones, encompassing heat shock cognate protein 70 (HSC70) and hereditary spastic paraplegia type 35 (HSP35), were substantially plummeted during autopsy of substantia nigra (SN) of individuals experiencing PD [79,80]. In addition, myriad genes have been expounded to partake in the ALP, for instance, *SNCA*, DJ-1, *GBA*, *LRRK2*, *PINK1*, *transmembrane protein 175* (*TMEM175*), *cathepsin B* (*CTSB*), *cathepsin D* (*CTSD*), *sphingomyelin phosphodiesterase 1* (*SMPD1*); however, mutations in these genes can culminate in the dysfunctioning of the ALP, and finally contributes to PD evolution [79,81,82,83]. Pursuant to another study, the clumping of α-synuclein and tau is triggered by abnormal autophagic lysosomal breakdown [81]. These aforementioned corroborations profoundly imply that ALP dysfunction significantly contributes to the pathogenesis of PD.

#### 2.2.3. Ubiquitin–Proteasome System Dysfunction and Parkinson’s Disease

The available literature has revealed that abnormal operation of the UPS is a prominent feature of multiple nerve cell deteriorating ailments, which are usually marked by impaired protein clumping. Autopsy analysis of the SN of PD individuals has displayed a noteworthy decline in the UPS biocatalyst operation compared to the brains of the healthy individuals, providing robustly solid corroboration for such aberrations in the case of PD [84]. Current publications have delineated the active engagement of mutations or alterations in several genes, viz., *UCHL1*, DJ-1, *Parkin*, *SNCA*, and *PINK1*, in prompting proteasomal irregularities, and consequently PD advancement [85,86]. In addition, it has been elucidated that an imperative ubiquitin E3 ligase, namely tumor necrosis factor receptor-associated factor 6 (TRAF6), is over expressed in the brains of individuals experiencing PD, and TRAF6 facilitates the trio, i.e., Lys6-, Lys27-, and Lys29-associated ubiquitination of α-synuclein and DJ-1, and in turn might precipitate the insoluble and polyubiquitinated mutant DJ-1 protein clumping. Further, autopsy analysis of the human brain with PD has displayed that the duo, namely α-synuclein and TRAF6, interact in identical spatial compartments, i.e., colocalize (inside the LBs) [85,87]. These examinations highlight the newfangled aptitude for TRAF6 and for aberrant ubiquitination in the pathology of PD. Another investigation has reported significant forfeiture of only α-subunits of 26 or 20S proteasomes inside DArgic nerve cells, disruption in the 20S proteasomal biocatalyst operations inside the SN-PC, and de-escalation in the quantities of proteasome activator 700 (PA700) and proteasome activator 28 (PA28) within the SN-PC of individuals experiencing PD [88]. These studies strongly suggest the active engagement of UPS dysfunction in the pathology of PD.

#### 2.2.4. Mitochondrial Devastation and Parkinson’s Disease

Existing work has disclosed that mitochondrial devastation is actively indulged in the PD advancement. Recently, it has been elucidated that abnormal expansion and fragmentation of the existing power plants of the cell (mitochondrial biogenesis), flawed breakdown of disrupted and redundant mitochondria (mitophagy), impaired electron transport chain (ETC) operation, escalated formation of ROS, abnormal trafficking, disrupted calcium equilibrium, alterations in the incessant processes of mitochondrial merging (mitochondrial dynamics), and, presumably, additional concomitant processes that significantly impact on the operation of mitochondria can all partake in the PD-related mitochondrial devastation [89,90]. Aside from producing an organic, energy-rendering molecule termed ATP, the power plants of cells also share their significant involvement in the modulation of calcium equilibrium, cellular demise via programmed cell death, generation and conveyance of Fe-sulphur(S) clusters, haem generation, and cellular expansion and fragmentation, which have all been demonstrated to be drastically altered in different forms of PD [89,91]. Moreover, environmentally-precipitated PD might emerge following the subjection to deleterious constituents, for instance, MPTP, rotenone, and paraquat, that consequentially suppress ETC (principally via suppressing the mitochondrial complex-I operation) [90,92]. Present-day exploration has demonstrated that alterations in numerous genes, viz., *SNCA*, *PINK1*, *LRRK2*, *Parkin*, DJ-1, and HTRA2, might elicit mitochondrial devastation and ultimately culminate in PD emergence [93]. These investigations highly indicate the critical participation of mitochondrial devastation in PD pathology.

#### 2.2.5. Apoptosis, Nerve Cell Inflammation, and Parkinson’s Disease

Apoptosis and nerve cell inflammation are regarded as the cardinal players in the PD pathology. Autopsy examination of the brain of individuals with PD have displayed the duo, namely autophagy and apoptosis [94]. In addition, it has been elucidated that a tiny class of inducible transcription factors, designated as Nuclear Factor kappa-light-chain-enhancer of activated B cells (NF-κB), has escalated inside the DArgic cells of PD patients [95]. Moreover, apoptosis and inflammation-associated events within the encephalon of PD-experiencing individuals are corroborated by numerous episodes, encompassing elevated quantities of the p53 gene (tumor suppressor), an inflammatory mediator denominated as interferon-gamma (IFN-γ), NF-ΚB, caspase activation within the SN, and alteration in the proapoptotic gene operation [96,97,98,99]. In accordance with another investigation, microglia stimulation was noticed inside the SN of PD-experiencing individuals, inevitably culminating in the liberation of proinflammatory molecules, encompassing interferons (IFNs), tumor necrosis factor-alpha (TNF-α), and interleukins (ILs), which, as a consequence, contributes to apoptosis in PD [59,100]. Likewise, α-synuclein clumping as well stimulates microglia, culminating in the protracted and insidious neuronal deterioration within the SN of PD-experiencing individuals [101]. Despite the fact that the processes eliciting PD-related microgliosis are nebulous, a dark brown colored pigment pinpointed within the cells, denominated as neuromelanin-embracing DA-producing neurons, was demonstrated to be profoundly vulnerable to the inflammation-related episodes in the malady. Still, it is elusive whether inflammatory events or episodes taking place in the vicinity of nerve cells are the fundamental culprit of PD or just a repercussion of the ailment.

#### 2.2.6. α-Synuclein Clumping and Parkinson’s Disease

Pursuant to published data, the two, namely erroneous folding and clumping of proteins, are integral pathogenic hallmarks of nerve cell deteriorating maladies [102]. Another investigation has revealed that the procurement of α-synuclein’s baneful operation, instead of forfeiture of its usual operation, is regarded as the causal factor of PD [103]. The α-synuclein accumulation, which contributes to its clumping, is profoundly associated with the procurement of the pernicious operation of α-synuclein. Moreover, erroneously folded α-synuclein accumulation is greatly exacerbated by alterations in the expression of a gene termed *SNCA*, which are probably precipitated by the trio, i.e., gene mutations, gene duplication, and gene triplication. This occurrence tremendously encouraged the scientists to perform an exhaustive investigation into the in vitro mechanism of α-synuclein clumping. Therefore, these findings probably bolster the speculation that the early-commencement form of PD is provoked by the expeditious fibril formation (fibrillation) of α-synuclein [104,105]. In addition, recombinant synthetic α-synuclein has the capability to effectuate clumping in vitro so as to bring about the fibrillation process, correspondingly to those pinpointed in vivo [106]. According to another investigation, the preformed fibrils (PFF) might disseminate in in vitro nerve cell culture in a “prion-like” fashion, and when administered directly inside the brain of a mouse by means of injection, they might disseminate in vivo in a similar fashion, culminating in the generation of pSer129-α-synuclein-positive LBs-like clumps, and eventually contribute to the emergence of PD [107]. Aside from clumping, α-synuclein enormously impacts tyrosine 3-monooxygenase/tyrosine hydroxylase (TH), autophagosome protein, HSP, and ubiquitin [102]. Additionally, it has been displayed that the different positions for ubiquitination may induce distinct consequences on α-synuclein clumping [108]. Moreover, it has been revealed that LBs-like α-synuclein clumps precipitate disruption in the entire macroautophagy via de-escalating the clearance of autophagosomes, which, as a consequence, culminates in escalated cellular demise [109]. Despite the fact that an escalating amount of corroborations strongly indicate that α-synuclein clumping is disrupted in PD, the definitive implication in the pathology of the malady is still elusive and mystified, and more investigation is required to clearly examine its contribution in the PD pathogenesis.

## 3. Deciphering the Neuroprotective Role of Neuropeptides in Parkinson’s Disease

**Figure 3:**
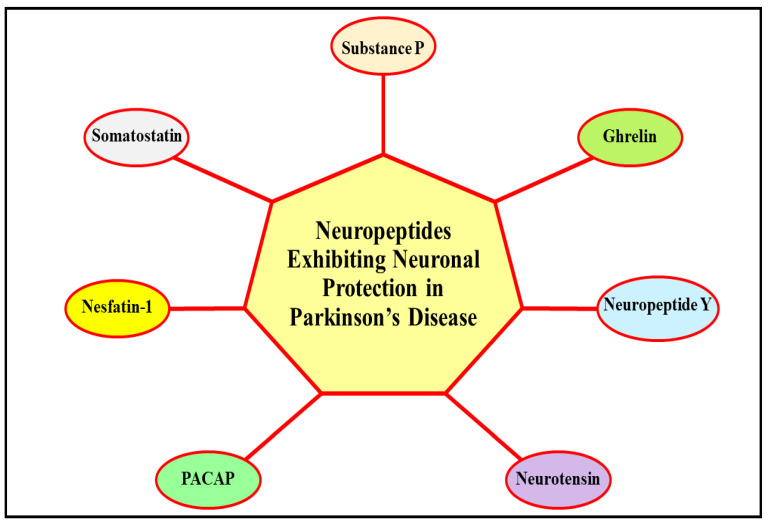
Highlighting the neuropeptides that contribute to significant nerve cell protection in Parkinson’s disease, encompassing substance P, ghrelin, neuropeptide Y, neurotensin, PACAP, nesfatin-1, and somatostatin. PACAP, pituitary adenylate cyclase-activating polypeptide.

Existing investigations have promulgated that NPs exert significant neuroprotective action in various PD models via inhibiting the pivotal pathways/mechanisms/processes involved in the evolution of the malady, and which are expounded in detail in the following sub-sections. Figure 3 describes the NPs that exhibit neuroprotective action in PD.

### 3.1. Neuroprotective Role of Substance P in Parkinson’s Disease

Substance P, an NP pertaining to one of the elephantine and eminently recognized peptide families, termed tachykinin (TK)/neurokinin (NK), comprehends eleven amino acid units and was first originated nearly nine decades ago by a Swedish pharmacologist named Ulf Svante von Euler and an English pharmacologist named Sir John Henry Gaddum [110]. The amino acid sequence of SP is as follows: H-Arg-Pro-Lys-Pro-Gln-Gln-Phe-Ple-Gly-Leu-Met-NH_2_ (Table 1) [111,112]. SP is enciphered by a protein coding gene pinpointed on human chromosome 7, namely the tachykinin precursor 1 gene (TAC1), and is known to provoke intestinal smooth muscle contraction [103]. It has been promulgated that SP is distributed in numerous regions of the human body, encompassing the encephalon, spinal column, intrinsic nervous system (INS)/enteric nervous system (ENS), largest body organ (skin), blood circulating vessels, and peripheral sensory nerves. In addition to SP, two more allied NPs comprising ten amino acid units, namely neurokinin A (NKA)/substance K and neurokinin B (NKB)/neuromedin K, have been spotted, and in conjunction, these NPs represent the TK family [113]. The trio, namely SP, NKA, and NKB, arise via the splitting of a precursor protein termed preprotachykinin, and each one of them behaves as a chemical messenger (neurotransmitter)/neuromodulator inside the CNS and the periphery. Even though the trio possesses identical functions, SP is still regarded as an ascendant member of the family. Most notably, SP is recognized to exhibit varied bodily functions by means of its interaction with three distinct categories of GPCRs/heptahelical receptors, namely neurokinin 1 (NK1), neurokinin 2 (NK2), and neurokinin 3 (NK3) [114,115].

Nearly four decades ago, a conducted investigation reported a consequential plummet in the SP-like immunoreactivity inside the nigral area and the outer compartment of the paleostriatum in individuals experiencing PD [116], which was subsequently corroborated by another investigation [117]. Another study elucidated individuals experiencing trouble in the pharynx while deglutition (pharyngeal dysphagia) demonstrated considerably de-escalated SP quantities in a body fluid termed spit/saliva, which is generated to a large extent in the salivary glands, in comparison to individuals experiencing PD with usual pharyngeal deglutition performance [118]. In addition, several investigations have detected alterations in the SP levels in 6-OHDA-instigated PD experimental models of rats. An investigation conducted around 33 years ago has revealed that the denervation of DA substantially de-escalated the SP levels inside the duo, namely the SN and the striate nucleus, following 21–28 days of 6-OHDA lesion [119]. In contrast, another investigation, conducted nearly a decade ago, has indicated that following 3–21 days of 6-OHDA therapy, quantities of SP were found to be considerably escalated inside the SN region of the brain [120]. This indicated that the 6-OHDA lesion precipitated a significant upsurge in SP quantities at first, and afterwards declined [121].

Up to the present time, the outcomes of SP therapy in PD are still considered tendentious. A recent investigation utilized a 1-methyl-4-phenylpyridinium ion(MPP^+^)-subjected DArgic nerve cell line (MES23.5 cells) to highlight the nerve cell safeguarding abilities of SP (when introduced at a 0.1 µM concentration) in cellular models of PD [30]. This investigation has displayed that SP by means of the NK1 receptor significantly safeguarded the MES23.5 cells against MPP^+^-precipitated apoptosis and cytotoxicity via de-escalating the entry of calcium ions, caspase-3 stimulation, ROS formation, and modulating the mitochondrial membrane potential (MtMP) [30]. Contrariwise, several investigations have elucidated that SP introduction consequentially elicits the DArgic nerve cell demise in PD models. To illustrate, the further introduction of SP in the 6-OHDA-prompted PD model escalated the evolution of the malady, with animals exhibiting intense motor abnormalities and worsened DArgic cell demise [120]. Another investigation has elucidated that therapy with the aid of 6-OHDA in mesostriatal organotypic coculture significantly escalated the levels of the duo, namely SP, and a biocatalyst and cellular demise indicator, named lactate dehydrogenase (LDH), thereby worsened the cellular demise [122]. Moreover, this investigation has reported that the generation of LDH was further skyrocketed following the integrated administration of SP and 6-OHDA but was plummeted following the integrated administration of 6-OHDA and an NK1 receptor antagonist named N-acetyl-L-tryptophan (NAT) [122].

**Figure 4:**
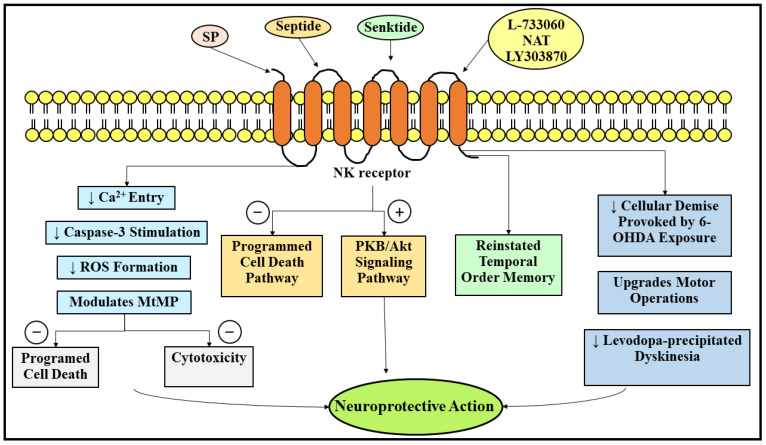
Highlighting the neuroprotective role of Substance P in Parkinson’s disease. SP upon interaction with NK1 receptor, decreases Ca^2+^ entry, caspase-3 stimulation, ROS formation, and modulates MtMP, which in turn inhibit programmed cell death and cytotoxicity, and thereby protects MES23.5 cells from MPP^+^-prompted neurotoxicity. Septide, an analog of SP, upon interaction with NK1 receptor culminates in the suppression of programmed cell death pathways and stimulation of PKB/Akt signaling pathway, thereby protects nerve cells against 6-OHDA-prompted neurotoxicity. Further, senktide, upon interaction with NK3 receptor, reinstated the temporal order memory in the 6-OHDA-lesioned hemiparkinsonian rat model. In addition, the three NK1 receptor antagonists, namely NAT, L-733060, and LY303870, upon introduction, decrease cellular demise provoked by 6-OHDA subjection, upgrade motor operations, and decrease levodopa-precipitated dyskinesia. Finally, by virtue of these mechanisms, SP markedly contributes to neuroprotective action in PD. Subtraction symbol indicates inhibitory/suppressing action, while addition symbol indicates stimulatory action. SP, Substance P; NAT, N-acetyl-L-tryptophan; LY303870, lanepitant; NK, neurokinin; NK1, neurokinin 1; NK3, neurokinin 3; Ca^2+^, calcium ions; ROS, reactive oxygen species; MtMP, mitochondrial membrane potential; MES23.5, DArgic nerve cell line; MPP^+^, 1-methyl-4-phenylpyridinium ion; PKB/Akt, protein kinase B signaling pathway; 6-OHDA, 6-hydroxy DA; PD, Parkinson’s disease; ↓, decreasing or reducing.

In the same way, the duo, namely agonists of the SP receptor and antagonists of the SP receptor, exhibited significant nerve cell protective actions in PD. A selective agonist of the NK1 receptor or an analog of SP, namely septide, has shown considerable nerve cell protective action against 6-OHDA-prompted pernicious repercussions following twenty-four hours of pretherapy at a 2 μM concentration via the suppression of the programmed cell death pathways and the stimulation of the protein kinase B (PKB/Akt) pathways (signal transduction pathways) [123]. Most notably, the antiprogrammed cell death action of septide was not reliant on caspase, which is congruent with another published paper highlighting the calpain-1-reliant nerve cell protective action of SP inside the cerebellar granule cells [124]. In addition, another exploration has elucidated that a selective agonist of NK3 receptor, namely senktide, following its administration in a dose of 0.2 mg/kg, reinstated the temporal order memory in the 6-OHDA-lesioned hemiparkinsonian rat model [125]. Howbeit, the intracerebroventricular introduction of the two neoteric, potent, and selective antagonists of the NK1 receptor, namely L-733060 and NAT, as well de-escalated the cellular demise provoked by 6-OHDA exposure, and finally contributed to a noteworthy upgradation in the motor operations [120]. In addition, another investigation has promulgated that NAT and another selective, potent, and neoteric antagonist of NK1 receptor, namely lanepitant (LY303870), markedly de-escalated the levodopa-precipitated anomalous, not controllable, and involuntary movements of muscles (dyskinesia), without influencing the promising medicinal outcomes of levodopa in animal rat models experiencing PD [126,127]. Furthermore, it has been revealed that the immune cells of the CNS, termed microglia, imitate the operation of professional phagocytes, termed macrophages, inside the encephalon. Additionally, it has been reported that the density of microglia inside the SN-PC region of the encephalon is markedly greater in comparison to the circumjacent regions of the encephalon [128]. Published literature has displayed that SP may be in part blameworthy for the greater microglia density. The microglia density in the SN region was markedly de-escalated in animal mice models lacking endogenous SP (TAC1^−/−^) or NK1 receptor (NK1R^−/−^) [118]. Moreover, this investigation has highlighted that SP captivated the microglia by means of a trio, namely NK1 receptor, protein kinase C delta (PKCδ), and reduced nicotinamide adenine dinucleotide phosphate (NADPH) oxidase in a pathway-reliant way. Figure 4 portrays the neuroprotective role of SP in PD.

### 3.2. Neuroprotective Role of Ghrelin in Parkinson’s Disease

Ghrelin, an inimitable gastric peptide/hunger hormone, comprehending twenty-eight amino acid units, was originally uncovered nearly 23 years ago by a Japanese researcher named Dr. Masayasu Kojima and fellow workers, and is chiefly liberated from the unfed stomach, but as well pinpointed in the tissues of the peripheral region, for instance, the ovary (a female gonad), testicle (a male reproductive gland), kidney, lymphocytes, pancreas (a mixed gland), placenta (a nonpermanent huge pan-shaped fetal organ that evolves in the course of pregnancy), pituitary (a master gland), and small bowel [129,130]. The amino acid sequence of ghrelin is as follows: NH_2_-Gly-Ser-[Ser(n-octanoyl)]-Phe-Leu-Ser-Pro-Glu-His-Gln-Arg-Val-Gln-Gln-Arg-Lys-Glu-Ser-Lys-Lys-Pro-Pro-Ala-Lys-Leu-Gln-Pro-Arg-COOH (Table 1) [131]. Ghrelin, initially spotted in the rat stomach, exists as an endogenous ligand of the rhodopsin-like or category A GPCRs, namely growth hormone (GH) secretagogue receptor 1a (GHS-R1a)/ghrelin receptor (ghrelinR), which is principally pinpointed in the encephalon and tissues of the peripheral region [130,132]. Ghrelin, upon interaction with its receptor, i.e., GHS-R1a, is able to stimulate the liberation of GH from adenohypophysis in order to elevate the concentration of calcium ions within the cells by means of the inositol 1,4,5-trisphosphate (InsP_3_) signaling pathway [123]. Ghrelin arises through the peptidal bond cleavage (proteolytic breakdown) of the two, namely proghrelin and preproghrelin [132]. Two significant types of ghrelin have been recognized in the liquid connective tissue (blood), namely acyl-ghrelin and non-acyl-ghrelin, and amongst the two, acyl-ghrelin has the aptitude to interact with GHS-R1a in order to exhibit physiological consequences [130]. In accordance with published literature, the acylated type of ghrelin, i.e., acyl-ghrelin, markedly renders nerve cell protection in PD [133,134,135].

It has been revealed that a consequential plummet in the concentrations of the duo, namely ghrelin and ghrelinR, are cognized to partake in the PD pathogenesis. Pursuant to a recent study, individuals experiencing PD display a consequential de-escalation in the fasting concentrations of the two, namely acyl-ghrelin (active type) and the entire ghrelin, which is further accompanied by a remarkable decline in the active type, in comparison to the salubrious control individuals [136]. Another investigation has reported that in experimental animal models with PD, the genetic deletion of GHSR significantly elevated the forfeiture of DA-forming nerve cells of the SN region of the encephalon, and consequently plummeted the DA concentrations in the striatal region, which may be turned back following the selective restimulation of GHSRinside the catecholaminergic (CArgic) nerve cells [137]. In addition, it has been promulgated that the introduction of a greatly employed ghrelinR antagonist, namely [D-Lys3]-GHRP6, through the microinjection/intracerebroventricular route within the SN region of a healthy experimental mice model might provoke PD-analogous motor coordination impairment [138].

Furthermore, an investigation conducted around 14 years ago initially demonstrated ghrelin’s nerve cell protective action in the MPTP-instigated experimental mouse model with PD [139], which was thereafter corroborated by numerous investigations [137,140,141]. It has been elucidated that ghrelin acted against or restrained cellular deprivation precipitated by exposure to a pesticide, i.e., rotenone [142,143], upgraded the abnormal rotarod motor performance in the experimental PD mouse model precipitated by subjection to MPTP [133], as well as mediated the nerve cell protective abilities of a nutritional strategy that markedly de-escalates the ingestion of calories without malnourishment, i.e., caloric restriction (CR) [144]. In addition, it has also been demonstrated that ghrelin has the aptitude to carry out the electrical stimulation of DA-forming nerve cells by suppressing the potassium (K^+^) channels (KCNQ/Kv7) and A-type voltage-gated K^+^ channels, as well as pursue the up-regulation of pacemaker channels/hyperpolarization-activated cyclic nucleotide-gated (HCN) channels so as to upgrade MPP^+^ suppression on DA nerve cell excitation [145,146]. In addition, it has been recently discovered that therapy with the aid of ghrelin contributed to a substantial escalation in the amount/number of neural stem cells (NSCs) of the midbrain, facilitated in vitro as well as ex vivo DArgic nerve cell differentiation via the canonical Wnt signaling pathway, and thereupon renders the prospect that subjection to ghrelin may be a newfangled, effective, and propitious strategy for PD therapy [147]. Another investigation has revealed that persistent exposure to a neoteric, and brain-permeable agonist of ghrelinR, namely HM01, was recognized to mitigate the nonmotor manifestations provoked by the 6-OHDA lesion in an experimental rat model experiencing PD, comprehending changes in the ingestion of water (H_2_O), consumption of food, weight of excrement, and body weight [148]. Moreover, Dpr3ghr, an analog of ghrelin, has been reported to safeguard human neuroblastoma (SH-SY5Y) cells (by escalating the expression of B-cell lymphoma-2 (Bcl-2), and de-escalating the Bcl-2-associated X protein (Bax) expression and Bax/Bcl-2 ratio) from the enormously perilous two carbonyl (C=O) groups comprising the organic compound, possessing the molecular formula CH_3_C(O)CHO, namely methylglyoxal (MGO)-precipitated programmed cell death and nerve cell toxicity [149].

**Figure 5:**
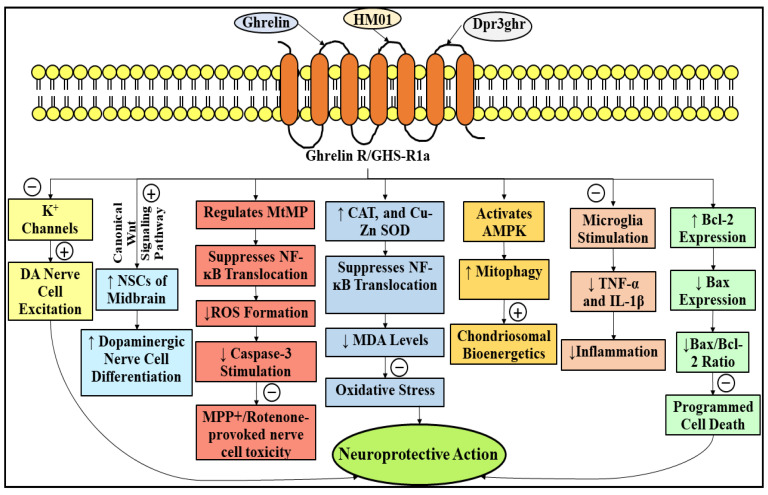
Highlighting the neuroprotective role of ghrelin in Parkinson’s disease. Ghrelin, along with its analogs, namely HM01 and Dpr3ghr, markedly render nerve cell protective actions against MPTP, MPP^+^, and rotenone-provoked neuronal destruction, via suppressing oxidative stress, programmed cell death, and de-escalating inflammation. Ghrelin, following its interaction with ghrelinR/GHS-R1a, suppresses K^+^ channels, which, as a consequence, contributes to DA nerve cell excitation. In addition, ghrelin, by means of the canonical Wnt signaling pathway, increases the number or amount of NSCs of the midbrain and in vitro and ex vivo DArgic nerve cell differentiation. Moreover, ghrelin treatment regulates MtMP, suppresses mitochondrial complex-I operation, de-escalates ROS formation and caspase-3 stimulation, and eventually restrains nerve cells from MPP^+^ or rotenone-instigated detrimental repercussions. Additionally, ghrelin escalates the levels of antioxidant biocatalysts, namely CAT and Cu-Zn SOD, de-escalates the MDA levels, and suppresses the NF-κB translocation, and thereby suppresses the oxidative stress via ceasing the lipid peroxidation and generation of ROS, and finally contributes to nerve cell protective action. Moreover, ghrelin possesses its nerve cell protective action via activating AMPK and escalating the mitophagy, which subsequently culminates in the amplification in the chondriosomal bioenergetics. Furthermore, therapy with the assistance of ghrelin culminates in the suppression of microglia stimulation, which, as a result, de-escalates inflammation (via de-escalating the levels of TNF-α and IL-1β), and finally culminates into nerve cell protective action. Apart from this, the two, namely ghrelin and Dpr3ghr, suppress programmed cell death and possess substantial nerve cell protective action by elevating Bcl-2 expression, and declining Bax expression and the Bax/Bcl-2 ratio. Subtraction symbol indicates inhibitory/suppressing action, while addition symbol indicates stimulatory action. GhrelinR, ghrelin receptor; GHS-R1a, growth hormone secretagogue receptor 1a; K^+^, potassium; DA, dopamine; NSCs, neural stem cells; DArgic, dopaminergic; MtMP, mitochondrial membrane potential; ROS, reactive oxygen species; MPP^+^, 1-methyl-4-phenylpyridinium ion; CAT, catalase; Cu-Zn SOD, copper-zincsuperoxide dismutase; NF-κB, Nuclear Factor kappa-light-chain-enhancer of activated B cells; MDA, malondialdehyde; AMPK, 5′ adenosine monophosphate-activated protein kinase; TNF-α,tumor necrosis factor-alpha; IL-1β, interleukin-1β;Bcl-2,B-cell lymphoma-2; Bax, Bcl-2-associated X protein; MPTP, 1-methyl-4-phenyl-1,2,3,6-tetrahydropyridine; ↑, increasing; ↓, decreasing.

The pathways by means of which ghrelin exerts its nerve cell protective actions are tortuous [150]. An early investigation, which employed a subacute MPTP-precipitated experimental model of mouse with PD, has indicated that the nerve cell protective outcomes of ghrelin may be strongly associated with a substantial plummet in the caspase-3-elicited programmed cell death through the modulation of the expression of the two, namely Bax and Bcl-2, within the DA-forming nerve cells of the nigral region of the encephalon [130]. Additionally, it has been revealed that ghrelin acted against MPP^+^ and rotenone-provoked nerve cell toxicity inside the MES23.5 cells and the principal retinal output cells, named retinal ganglion cells (RGCs), via regulating the MtMP, eradicating the formation of ROS, and suppressing the mitochondrial complex-I operation and stimulation of caspase-3 [142,143,151]. The nerve cell protective action rendered by ghrelin is markedly reliant upon the duo, namely chondriosomal biogenesis and chondriosome-associated oxidative damage [121]. It has been propounded that a chondriosomal protein, namely uncoupling protein 2 (UCP2)-reliant changes in two, i.e., chondriosomal respiration and bio-energetic status purveying, prompted by subjection to ghrelin, may construct the DA-forming nerve cells immensely invulnerable to cell destruction [137]. Moreover, it has been expounded that ghrelin has exhibited its actions against oxidative stress by consequentially escalating the operation of two, namely CAT and Cu-zinc (Zn) SOD, suppressing the translocation of NF-κB, and plummeting the MDA levels [152]. In addition, it has been revealed that the nerve cell protective action provoked by ghrelin exposure was enormously reliant upon an imperative energy sensor, i.e., 5′ adenosine monophosphate-activated protein kinase (AMPK), and substantially escalated the devastation of the deteriorated chondriosome (mitophagy) inside the DA-generating nerve cells [153]. Another study has elucidated that in an MPTP-subjected experimental PD mouse model, ghrelin might possess its nerve cell protective outcomes via suppressing the microglia stimulation, which, as a consequence, suppresses the liberation of a couple of inflammatory mediators, viz., TNF-α and interleukin-1β (IL-1β) [141]. Figure 5 describes the neuroprotective role of ghrelin in PD.

### 3.3. Neuroprotective Role of Neuropeptide Y in Parkinson’s Disease

NPY, a prolific and foremost NP, initially originated around four decades ago from the pig encephalon by a well-renowned researcher named Kazuhiko Tatemoto and fellow workers, pertains to a category of three thirty-six amino acid units comprising peptides, and the remaining two, i.e., peptide YY (PYY) and pancreatic polypeptide (PP), exist as gastrointestinal hormones [154,155]. The amino acid sequence of NPY is as follows: Tyr-Pro-Ser-Lys-Pro-Asp-Asn-Pro-Gly-Glu-Asp-Ala-Pro-Ala-Glu-Asp-Leu-Ala-Arg-Tyr-Tyr-Ser-Ala-Leu-Arg-His-Tyr-Ile-Asn-Leu-Ile-Thr-Arg-Gln-Arg-Tyr-NH_2_ (Table 1) [156]. NPY is profusely pinpointed in the peripheral region and the varied regions of the encephalon, such as the nucleus tractus solitarius, hypothalamus, hippocampus, cerebral mantle, accumbens nucleus, amygdala (the fear center of the encephalon), and locus coeruleus (LC) [155,157]. NPY has been elucidated to interact with NPY receptors that pertain to the rhodopsin-like or category A GPCRs [158]. Up to the present day, five receptors of NPY, namely Y_1_ receptor (Y_1_R), Y_2_ receptor (Y_2_R), Y_4_ receptor (Y_4_R), Y_5_ receptor (Y_5_R), and Y_6_ receptor (Y_6_R), displaying nonidentical operations, have been cloned and recognized from mammals, and multiple other receptors, for instance, Y_3_ receptor (Y_3_R), and have been speculated (relying upon their therapeutic aptitude) to employ numerous animal and human tissues, but were not cloned or clearly recognized until now [155,158,159]. Amongst the five, i.e., Y_1_R, Y_2_R, Y_4_R, Y_5_R, and Y_6_R, only Y_6_R is operational in animals, for instance, in mice and rabbits, whereas the rest of the receptors are operational in human beings [158,160]. In addition, NPY receptors fundamentally bind with G inhibitory (G_i_)/G_0_ proteins and culminate in the suppression of adenyl cyclase (AC), and eventually contribute to the suppression of the build-up of cyclic adenosine monophosphate (cAMP) and the regulation of the two, i.e., K^+^ channels and calcium channels. Moreover, a pair of NPY receptors, namely Y_2_R and Y_4_R, as well binds to a protein termed G_q_ protein, culminating in the escalated formation of InsP_3_ through phospholipase C-beta (PLC-β) stimulation inside the smooth muscle cells of the rabbit [158]. Published work has expounded that NPY significantly participates in the regulation of several operations, for instance, the consumption of food, learning (procurement of novel knowledge and skills, or flourishing the one known previously), mood (a conscious mind state), memory (procurement, storage, and retrieval of information) [161], and nerve cell protection against nerve-cell-deteriorating ailments [162].

Numerous investigations entailing animal models and human beings have demonstrated substantial alterations in the levels of NPY. Around 31 years ago, an investigation involving individuals experiencing PD displayed markedly de-escalated levels of NPY in their tissues of the adrenal medulla [163]. After about a year, another research team evaluated the levels of NPY immunoreactivity within the fluid encircling the encephalon and the spinal column, termed cerebrospinal fluid (CSF), of 10 individuals suffering from PD, and concluded that the NPY levels were consequentially plummeted, comparably to healthy subjects, reflecting a marked decrease in the liberation of NPY or a marked elevation in the turnover of NPY [164]. Furthermore, an autopsy examination of the encephalon samples of the PD individuals has displayed an upsurge in the amount of NPY messenger ribonucleic acid (mRNA)-positive cells within their trio, namely accumbens nucleus, caudate nucleus, and putamen [165]. Another investigation has demonstrated a considerable decline in the NPY-positive cells, as well as the deprivation of axons within the two, namely caudate nucleus and putamen, of individuals experiencing lubag syndrome/X-linked dystonia of panay [166]. Moreover, individuals with lubag syndrome/X-linked dystonia of panay have indicated the paucity of the labeling of NPY within the subventricular zone (SVZ), together with a consequential forfeiture of proliferating cell nuclear antigen (PCNA)-representing progenitor cells (PGCs) [166]. Another group of researchers have described that those individuals experiencing the duo, i.e., PD and depression at the same time, exhibited a marked upsurge in the NPY levels within their CSF in comparison to individuals experiencing depression alone [167]. In addition, the deterioration of the DArgic pathway, named the nigrostriatal pathway, provoked a prodigious upsurge in the NPY-like immunoreactivity within the striatal region of the MPTP-subjected experimental C57 black 6 (C57BL/6) mice model [168].

**Figure 6:**
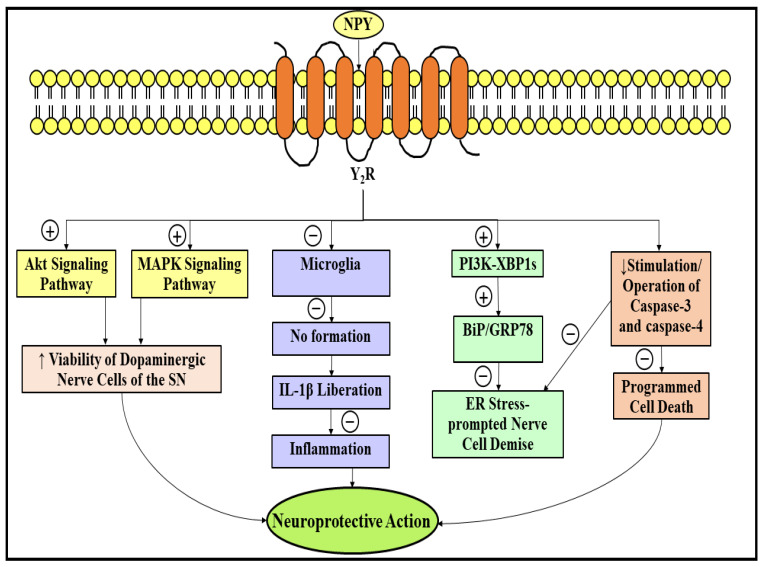
Highlighting the neuroprotective role of neuropeptide Y in Parkinson’s disease. NPY exhibits its significant nerve cell protective action upon interacting with DA-forming cells by means of Y_2_R. NPY stimulates the two, namely the Akt signaling pathway and the MAPK signaling pathway and contributes to the enhanced viability of DA-forming nerve cells of the nigral area of the encephalon. In addition, NPY exerts its nerve cell protective action by suppressing the microglia, which in turn suppresses the NO formation and IL-1β liberation, and finally results in the suppression of inflammation. Furthermore, NPY, via stimulating the PI3K-XBP1s-Bip/GRP78 signaling pathway, suppresses the ER stress-provoked nerve cell demise, and ultimately contributes to neuroprotection. Moreover, therapy with the assistance of NPY de-escalated the stimulation/operation of caspase-3 and caspase-4, which consequently suppressed the programmed cell death and ER stress-triggered nerve cell demise and contributed to nerve cell protection in PD. Subtraction symbol indicates inhibitory/suppressing action, while addition symbol indicates stimulatory action. NPY, neuropeptide Y; Y_2_R,Y_2_ receptor; DArgic, dopaminergic; SN, substantia nigra; MAPK, mitogen-activated protein kinase; NO, nitric oxide; IL-1β, interleukin-1β; PI3K, phosphoinositide 3-kinase; XBP1s, spliced form of X-box binding protein 1; BiP, binding immunoglobulin protein; GRP78, 78-kDa glucose-regulated protein; ER stress, endoplasmic reticulum stress; DA, dopamine; PD, Parkinson’s disease; ↑, increasing; ↓, decreasing.

It has been shown that NPY renders substantial nerve cell protection in PD through multifarious processes associated with the ailment. Pursuant to an investigation, the Y_2_R emanates to be an eminent and cardinal receptor of NPY, which is reported to be enormously liable for the mediation of nerve cell protective action of NPY, since the nerve cell protection rendered by NPY therapy was ceased in mice subjected to the Y_2_R antagonist and in mice lacking Y_2_R [169]. The nerve cell protective action of NPY was initially discovered in the duo, i.e., in vivo and in vitro 6-OHDA-precipitated experimental PD models [169]. Further, this investigation has strongly suggested that the nerve cell protective action of NPY is attained to a certain extent via the stimulation of two signaling pathways, namely Akt and mitogen-activated protein kinase (MAPK), which might culminate into robust enhancement in the viability of DA-forming nerve cells of the nigral area of the encephalon [169]. Recently, it has also been elucidated that in experimental rat models exposed to 6-OHDA, NPY treatment culminated in the suppression of microglia in two regions, i.e., striatal and SN, which, as a consequence, facilitated the anti-inflammatory action of NPY in the PD [170]. Furthermore, it has been reported that the subjection to NPY resulted in the suppression of an imperative constituent of the gram-negative bacteria, termed lipopolysaccharide (LPS)-triggered NO formation, and the liberation of IL-1β inside the microglia [171]. In accordance with another exploration, NPY treatment, via pursuing the stimulation of the phosphoinositide 3-kinase (PI3K)-spliced form of X-box binding protein 1 (XBP1s)-precipitated binding immunoglobulin protein (BiP)/78-kDa glucose-regulated protein (GRP78) pathway, markedly exhibited a safeguarding action against an endoplasmic reticulum stress (ER stress)-prompted nerve cell demise [172]. Additionally, the prior introduction of NPY significantly de-escalated the stimulation or operation of the duo, namely caspase-3 and caspase-4, in the duration of the ER stress reaction [172]. Figure 6 depicts the neuroprotective role of NPY in PD. In addition, a protein termed abrineurin/brain-derived neurotrophic factor (BDNF), which is implicated in the growth, differentiation, maintenance, and promotion of the viability of nerve cells, was presumed to be deeply entangled in bringing about the nerve cell protective action of NPY [173]. The forfeiture of DA-forming nerve cells of the SN in PD is speculated to be provoked by the plummeted BDNF expression [173,174]. To date, no literature exists that could clearly expound the putative association amongst the duo, namely NPY and BDNF expression, in PD. Therefore, more research is tremendously required in this particular domain to scrutinize the potential consequences of NPY on the expression of BDNF in PD.

### 3.4. Neurprotective Role of Neurotensin in Parkinson’s Disease

Neurotensin, an NP comprehending thirteen amino acid units, was initially originated nearly 49 years ago from the calf hypothalamus by two prestigious researchers, namely Robert Carraway and Susan E. Leeman, and denominated owing to its localization in the nerve cells and hypotensive action (blood pressure-decreasing aptitude) [175,176]. The amino acid sequence of neurotensin is as follows: pyr-Glu-Leu-Tyr-Glu-Asn-Lys-Pro-Arg-Arg-Pro-Tyr-Ile-Leu-OH (Table 1) [177]. This modulatory endogenous peptide is pinpointed in two regions, i.e., the CNS (principally the pituitary and hypothalamus) and the peripheral region (principally the gastrointestinal tract), where it behaves as a modulator/transmitter for nerve cells and locally as a hormone, respectively [178,179]. Currently, three receptors for neurotensin have been spotted, namely neurotensin receptor 1 (NTR1), neurotensin receptor 2 (NTR2), and neurotensin receptor 3 (NTR3), and neurotensin, upon interaction with these receptors, exhibits its bioactivities. The duo, namely NTR1 and NTR2, pertains to the rhodopsin-like or category A 7TM domain GPCRs, whereas the third one, namely NTR3, a category I single TM domain sorting receptor, otherwise designated as sortilin, pertains to the category of vacuolar protein sorting 10 protein (VPS10P) domain receptors [179,180]. Consequently, the actions of neurotensin enormously rely upon the two, i.e., the type of receptor and the dispersal inside the regions of the body.

Pursuant to the existing data, neurotensin is consequentially implicated in the DArgic pathway. In accordance with histological examinations of rat encephalon, ample neurotensin-comprising fibers have been detected in regions possessing plenteous DA content, for instance, SN and ventral tegmentum [181]. Further, two other investigations have displayed a bifold escalation in the neurotensin content inside the SN area of encephalon of PD-experiencing individuals [182,183]. In addition, it has been reported that individuals experiencing PD exhibited markedly escalated neurotensin content within their plasma in comparison to normal healthy individuals, and four nontreated individuals also exhibited significantly elevated neurotensin content within their plasma in comparison to individuals subjected to levodopa therapy [184].

In addition, the neurotensin receptor mRNA quantities were discovered to be markedly elevated within the rat encephalon DA-forming nerve cells of the duo, namely the SN and ventral tegmentum [185]. Howbeit, in PD-experiencing individuals, considerably plummeted quantities or zero/nil mRNA expression for neurotensin receptors were detected inside the ventral tier of the SN region of the encephalon [185]. Moreover, a consequential decline in the concentrations of neurotensin receptors was reported within the two regions, namely the paleostriatum and putamen of PD-experiencing individuals [186,187,188].

Published literature has proven that neurotensin and its analogs have the aptitude to render significant nerve cell protective action in PD-experiencing experimental animal models. It has been described that the introduction of the duo, i.e., neurotensin 8–13 and [D-Tyr11]-neurotensin, through the intracerebroventricular route consequentially de-escalated the 6-OHDA-precipitated PD manifestations (tremor, and rigor) [189]. Another investigation has displayed two neoteric analogs of neurotensin, namely neurotensin2 and neurotensin4, might amplify the liberation of DA within the striatal region, de-escalate the rotations provoked by exposure to an immensely potent agonist of DA, namely apomorphine, and upgrade memory and learning [34]. Howbeit, it has been elucidated that, via escalating the fundamental excitatory neurotransmitter (glutamate)-provoked nerve cell toxicity by elevating the calcium levels within the cells or/and N-methyl-D-aspartate (NMDA)-triggered signaling of glutamate, neurotensin elevated the deterioration of the two, viz., cortical nerve cells and DArgic mesencephalic nerve cells [190]. Therefore, more exploration is enormously needed in this respective discipline to elucidate the potential role of neurotensin in PD.

### 3.5. Neuroprotective Role of Pituitary Adenylate Cyclase-Activating Polypeptide in Parkinson’s Disease

PACAP, an enormously preserved and multitalented NP, comprehending twenty-seven amino acid units, i.e., PACAP27/thirty-eight amino acid units, i.e., PACAP38, was initially originated around 33 years ago from the extracts of sheep hypothalamus by a well-known endocrinologist named Akira Arimura and fellow workers, and pertains to the class of secretin-glucagon-vasoactive intestinal polypeptide (VIP) [191,192]. The amino acid sequence of PACAP27 is as follows: H-His-Ser-Asp-Gly-Ile-Phe-Thr-Asp-Ser-Tyr-Ser-Arg-Tyr-Arg-Lys-Gln-Met-Ala-Val-Lys-Lys-Tyr-Leu-Ala-Ala-Val-Leu-NH_2_, whereas the amino acid sequence of PACAP38 is as follows: H-His-Ser-Asp-Gly-Ile-Phe-Thr-Asp-Ser-Tyr-Ser-Arg-Tyr-Arg-Lys-Gln-Met-Ala-Val-Lys-Lys-Tyr-Leu-Ala-Ala-Val-Leu-Gly-Lys-Arg-Tyr-Lys-Gln-Arg-Val-Lys-Asn-Lys-NH_2_ (Table 1) [193,194]. PACAP is robustly engaged in the regulation of multiple biological operations within the CNS and peripheral region upon interaction with three category B GPCRs, otherwise designated as secretin class of GPCRs, namely PACAP type I receptor (PAC1), VIP receptor 1 (VPAC1), and VIP receptor 2 (VPAC2) [191,195]. Amongst the aforementioned three receptors, PACAP exhibits 10^3^-fold greater affinity towards PAC1 in comparison to the duo, namely VPAC1 and VPAC2 [185]. PACAP is reported to be enciphered by a gene named *adenylate cyclase activating polypeptide 1* (*ADCYAP1*) [196,197]. At elevated concentrations, PACAP is principally pinpointed in the accumbens nucleus, SN, hippocampus, cerebellum, hypothalamus, and the bed nucleus of the stria terminalis (BNST) [121,198]. Within the CNS, PACAP behaves as a neurotrophic factor, neurohormone, neuroregulator, and neurotransmitter [199].

In addition, it has been revealed that mRNA that is enormously implicated in enciphering PACAP receptors has been detected within the SN [199]. Another investigation has elucidated that in MPTP-instigated experimental macaque monkey PD models, a consequential plummet in the immunological signal of PAC1 receptor was recognized within various regions of the basal nuclei, encompassing the caudate nucleus, inner and outer regions of the paleostriatum, and putamen [199].

Numerous investigations have displayed the nerve cell protective abilities of PACAP in various experimental models experiencing PD [200,201]. It has been depicted that PACAP markedly declines the DArgic nerve cell deprivation provoked by 6-OHDA exposure, upgrades behavioral impairment [202], de-escalates the fundamental manifestation of PD (hypokinesia) [203], and slows the decline in DA content. PACAP might have the aptitude to forestall the impaired polypeptide chain generation precipitated by the subjection to MPTP and reduce cognitive deterioration [204]. Another investigation has reported that PACAP was significantly capable of de-escalating the programmed cell death and easing the conversion of cellular demise from the late to early phase in a rotenone-prompted cellular PD model [205]. Additionally, PACAP therapy has safeguarded the SH-SY5Y cells from a nerve cell active candidate, named 1-methyl-6,7-dihydroxy-1,2,3,4-tetrahydroisochinolin, precipitated deleterious consequences via consequentially decreasing the programmed cell death and the related chemical alterations [206]. In addition, it has been elucidated that PACAP27 as well markedly de-escalated the DArgic nerve cell deprivation and motor abnormalities in a prostaglandin J2-provoked experimental PD model [207].

Existing publications have demonstrated that PACAP renders substantial nerve cell protection in PD through multifaceted processes. Initially, the nerve cell protective action of PACAP was immensely related to its inflammation-reducing abilities. It has been reported that prior therapy of SH-SY5Y cells with a tremendously potent agonist of the PACAP receptor, namely PACAP (1–38), significantly contributed to a dosage-reliant de-escalation of deleterious repercussions provoked by the mediators of inflammation [208]. Moreover, PACAP has been depicted to possess considerable antiautophagic abilities. A recent exploration has demonstrated that PACAP therapy considerably plummeted the autophagic operation in MPTP-provoked experimental PD models via regulating the concentrations of a protein termed sequestosome-1/p62, and by carrying out the formation of microtubule-associated protein light chain 3-phosphatidylethanolamine conjugate (LC3-II) [195]. Another research group has revealed that the safeguarding action of PACAP against cellular demise precipitated by rotenone exposure was markedly suppressed through the introduction of suppressors of the trio, namely p38 MAPK, protein kinase A (PKA), and extracellular signal-regulated kinase (ERK) [205]. Consequently, the nerve cell protective actions of PACAP were attained via the stimulation of PKA signaling process, and also the two downstream signals, i.e., p38 MAPK and ERK [205]. This nerve cell protective action was cognized to be immensely linked to a balance among DA-acetylcholine (ACh) processes within the nerve cell pathway of the basal nuclei. Moreover, it has been elucidated that the introduction of PACAP27 through the intravenous route in an MPTP-precipitated experimental mouse model with PD rendered significant nerve cell protective action via altering the DArgic, as well as cholinergic synaptic conveyance, by means of escalating the operation of DA 2 receptors (D2R) and the expression of ATP-sensitive potassium channel (KATP) subunits within the striatal region of the basal nuclei [209]. Furthermore, therapy with the aid of PACAP/agonists of PACAP receptors culminated in the significant de-escalation in the SH-SY5Y cells toxicity provoked by 1-methyl-6,7-dihydroxy-1,2,3,4-tetrahydroisochinolin exposure via suppressing caspase-3 expression and elevating the expression of the duo, namely BDNF and phosphorylated cAMP-response element binding protein (p-CREB) [210]. In addition, the nerve cell protective action rendered by PACAP therapy is thought to be immensely linked to microglia. PACAP might have the aptitude to significantly reduce LPS-provoked microglia stimulation and the concomitant formation, as well as the liberation of NO and TNF-α, respectively [210]. Howbeit, another investigation has revealed that PACAP27 was incapable of restraining the stimulation of microglia in the prostaglandin J2-precipitated experimental PD mouse model [207]. As a result, further scrutiny is greatly required in this respective discipline to ascertain the clear participation of PACAP in the stimulation of microglia in PD.

**Figure 7:**
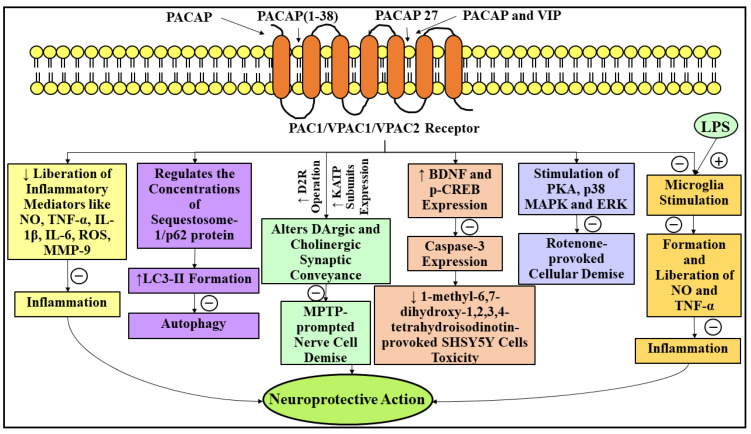
Highlighting the neuroprotective role of pituitary adenylate cyclase-activating polypeptide in Parkinson’s disease. The duo, namely experimental animal and cellular PD models illustrate that PACAP therapy renders significant nerve cell protective action against rotenone, MPTP, and 1-methyl-6,7-dihydroxy-1,2,3,4-tetrahydroisochinolin-provoked deleterious repercussions through suppressing inflammation, autophagy, and cellular demise. The prior therapy of SH-SY5Y cells with PACAP (1–38) safeguarded these cells against inflammatory-mediated detrimental effects via decreasing the liberation of inflammatory mediators. PACAP regulates the sequestosome-1/p62 protein concentrations and elevates the LC3-II formation, and thereby suppresses the autophagic-operation. PACAP safeguarded against rotenone-precipitated cellular demise via carrying out the stimulation of PKA signaling process, as well as the two downstream signals, viz., p38 MAPK and ERK. In addition, intravenously introduced PACAP27 safeguarded against MPTP-instigated nerve cell demise via altering the DArgic and cholinergic synaptic conveyance, by way of elevating the D2R operation and the KATP subunits expression within the striatal region of the basal nuclei. Further, therapy with the assistance of PACAP/agonists of PACAP receptors elevated the BDNF and p-CREB expression, and suppressed the caspase-3 expression, and, as a consequence, decreased the 1-methyl-6,7-dihydroxy-1,2,3,4-tetrahydroisochinolin-prompted SH-SY5Y cells toxicity. Apart from this, PACAP has the tendency to considerably suppress the LPS-prompted microglia stimulation, which in turn suppresses the formation and liberation of NO and TNF-α, and finally suppresses the inflammation. Subtraction symbol indicates inhibitory/suppressing action, while addition symbol indicates stimulatory action. PACAP, pituitary adenylate cyclase-activating polypeptide; VIP, vasoactive intestinal polypeptide; PAC1, PACAP type I receptor; VPAC1, VIP receptor 1; VPAC2, VIP receptor 2; NO, nitric oxide; TNF-α, tumor necrosis factor-alpha; IL-1β, interleukin-1β; IL-6, interleukin-6; ROS, reactive oxygen species; MMP-9, matrix metallopeptidase 9; LC3-II, microtubule-associated protein light chain 3-phosphatidylethanolamine conjugate; PKA, protein kinase A; MAPK, mitogen-activated protein kinase; ERK, extracellular signal-regulated kinase; D2R, DA 2 receptors; KATP, ATP-sensitive potassium channel; DArgic, dopaminergic; MPTP, 1-methyl-4-phenyl-1,2,3,6-tetrahydropyridine; BDNF, brain-derived neurotrophic factor; p-CREB, phosphorylated cAMP-response element binding protein; SH-SY5Y, human neuroblastoma cells; LPS, lipopolysaccharide; PD, Parkinson’s disease; ↑, increasing; ↓, decreasing.

VIP, an NP identical to PACAP, comprises twenty-eight amino acid units and was initially derived from porcine duodenum nearly 52 years ago by two renowned researchers, namely Sami I Said and Viktor Mutt [211]. Recently, a research group has elucidated that VIP exhibits the identical nerve cell protective actions as that of PACAP [212]. Another investigation has reported that VIP therapy restrained the MPTP-elicited deprivation of two, i.e., DArgic nerve cells and nerve fibers, within the nigrostriatal pathway via suppressing the liberation of inflammatory mediators, for instance, NO, ROS, IL-1β, and TNF-α [213]. In accordance with another recent investigation, PACAP and VIP cotherapy markedly restrained the liberation of NO, interleukin-6 (IL-6), matrix metallopeptidase 9 (MMP-9), and cluster of differentiation molecule 11b (CD11b) in the rotenone-subjected microglial cells of mice (BV-2 cells), and therefore might contribute to considerable nerve cell protection in PD [214]. Figure 7 represents the neuroprotective role of PACAP in PD.

### 3.6. Neuroprotective Role of Nesfatin-1 in Parkinson’s Disease

Nesfatin-1 is an inimitable and enormously effective appetite-suppressing NP, comprising eighty-two amino acid units procured from the three hundred and ninety-six amino acid units comprising a parent protein termed nucleobindin-2 (NUCB2), and was initially originated around 16 years ago from the hypothalamus of the rat by a Japanese researcher named Shinsuke Oh-I and fellow workers, and is extensively pinpointed in the duo, i.e., the CNS and the peripheral areas [215,216,217]. The amino acid sequence of nesfatin-1 is as follows: Val-Pro-Ile-Asp-Ile-Asp-Lys-Thr-Lys-Val-Gln-Asn-Ile-His-Pro-Val-Glu-Ser-Ala-Lys-Ile-Glu-Pro-Pro-Asp-Thr-Gly-Leu-Tyr-Tyr-Asp-Glu-Tyr-Leu-Lys-Gln-Val-Ile-Asp-Val-Leu-Glu-Thr-Asp-Lys-His-Phe-Arg-Glu-Lys-Leu-Gln-Lys-Ala-Asp-Ile-Glu-Glu-Ile-Lys-Ser-Gly-Arg-Leu-Ser-Lys-Glu-Leu-Asp-Leu-Val-Ser-His-His-Val-Arg-Thr-Lys-Leu-Asp-Glu-Leu (Table 1) [218]. It has been reported that within the encephalon, nesfatin-1 is primarily pinpointed in the nucleus tractus solitarius, master gland, infundibular nucleus, dorsal vagal nucleus, supraoptic nucleus, and hypothalamic paraventricular nucleus [219]. Moreover, nesfatin-1 might have the aptitude to permeate across the blood–brain barrier through nonsaturable pathways in two ways, i.e., blood to encephalon and encephalon to blood [220]. In addition, nesfatin-1 partakes in the modulation of the blood sugar equilibrium and the expenditure of energy, and also exhibits anti-inflammatory as well as antiprogrammed cell death abilities [208]. Despite emerging corroboration for the plethora of NP’s abilities, the GPCR that is implicated in the mediation of these actions remains obscure.

Pursuant to a recently published investigation, the blood concentration of nesfatin-1 in individuals experiencing PD was considerably plummeted in comparison to control individuals [221]. The nerve cell protective action of nesfatin-1 in PD might be owing to its aptitude to suppress inflammation, oxidative stress, and programmed cell death.

Several investigations have depicted the protective action of nesfatin-1 against inflammation in the encephalon. It has been reported that nesfatin-1 therapy markedly de-escalated the NF-κB expression and the concentrations of inflammatory mediators like IL-1β, TNF-α, and IL-6 in experimental rat models with intracranial injury, strongly indicating that nesfatin-1 may inhibit the NF-κB-reliant inflammatory reactions [222]. Furthermore, therapy with the aid of nesfatin-1 significantly suppressed the acute encephalon damage following the subarachnoid hemorrhage-prompted diffusion and build-up of neutrocytes and the escalated quantities of inflammatory mediators [223].

Another investigation has revealed that in MES23.5 DA nerve cells, nesfatin-1 therapy potentially recovered the suddenly falled MtMP provoked by subjection to rotenone, as well as reinstated the mitochondrial complex-I operation [224]. According to another recent exploration, nesfatin-1 introduction in the brain ischemia tremendously suppresses lipid peroxidation and escalates the operation of biocatalysts exhibiting antioxidant abilities, namely GSH and SOD [225]. Apart from this, nesfatin-1′s ability against oxidative destruction has also been depicted in subarachnoid hemorrhage models [223].

Further, prior therapy with nesfatin-1 markedly safeguards MES23.5 DA nerve cells from nerve cell toxicity precipitated by rotenone exposure via suppressing programmed cell death and improving abnormal mitochondrial operation [224]. Another research group has reported that the antiprogrammed cell death action of nesfatin-1 inside the DA-forming nerve cells was fundamentally attained via the C-Raf/ERK1/2-reliant antiprogrammed cell death mechanism, by means of which nesfatin-1 consequentially inhibits the caspase-3 operation, and finally contributes to the suppression of programmed cell death [226] In the same investigation, the PKA suppressor did not suppress the nesfatin-1 action, tremendously propounding the absence of engagement of the PKA pathway in rendering antiprogrammed cell death action [226].

By and large, the actual and circumstantial molecular pathways engaged in rendering the nerve cell protective action of nesfatin-1 yet remain unexplored. Despite the fact that the pathways implicated in bringing about nesfatin-1′s nerve cell protective action have been depicted in numerous experimental models experiencing varied sort of neurological maladies, it is still greatly inexplicit whether these nerve cell protective pathways are pertinent to PD. Consequently, an in-depth exploration is immensely required so as to attain corroborations for the employment of nesfatin-1 in therapeutic settings.

### 3.7. Neuroprotective Role of Somatostatin in Parkinson’s Disease

SST, otherwise designated as growth hormone-inhibiting hormone (GHIH), is a renowned cyclic ring structure containing modulatory NP, occurring in two active types, the first one comprises of fourteen amino acid units, while the second one comprises of twenty-eight amino acid units, and was initially originated nearly 48 years ago from the extracts of sheep hypothalamus by Paul Brazeau and fellow workers, and is fundamentally pinpointed in the duo, i.e., the CNS and the peripheral areas [227,228]. The amino acid sequence of fourteen amino acid units comprising SST is as follows: Ala-Gly-Cys-Lys-Asn-Phe-Phe-Trp-Lys-Thr-Phe-Thr-Ser-Cys, whereas the amino acid sequence of twenty-eight amino acid units comprising SST is as follows: Ser-Ala-Asn-Ser-Asn-Pro-Ala-Met-Ala-Pro-Arg-Glu-Arg-Lys-Ala-Gly-Cys-Lys-Asn-Phe-Phe-Trp-Lys-Thr-Phe-Thr-Ser-Cys (Table 1) [229]. SST is generated in numerous regions within the body, encompassing the CNS, digestive tract, hypothalamic region, and pancreas [230]. SST is reported to behave as a neurotransmitter, nerve cell regulator, and highly active suppressor of dysfunctional cellular multiplication and several secretory pathways, upon interaction with five 7TM domain GPCRs, viz., SST receptor 1 (SSTR1), SST receptor 2 (SSTR2), SST receptor 3 (SSTR3), SST receptor 4 (SSTR4), and SST receptor 5 (SSTR5) [231,232]. SST markedly exerts wide range of suppressing actions on the duo, namely exocrine secretion (such as pancreatic biocatalysts, stomach acid, and intestinal fluid), and endocrine secretion (such as VIP, GH, PP, cholecystokinin, glucagon, secretin, insulin, and gastrin) [228,231,233,234].

Up to the present time, only a single research group has explored the nerve cell protective abilities of SST in nerve cell deteriorating diseases, for instance, PD [37]. In this investigation, researchers have employed an LPS-subjected experimental PD rat model so as to examine the significant outcomes of SST therapy on DArgic nerve cell deterioration provoked by LPS exposure. Pursuant to this investigation, LPS exposure markedly contributed to DArgic nerve cell deprivation, whereas a consequential plummet in the nerve cell demise via SST prior therapy was strongly corroborated by means of immunohistochemical staining of the duo, i.e., TH- and Nissl-positive cells [37]. In addition, this study has reported that SST substantially suppressed the formation of the ROS and LPS-provoked microglia operation [37]. Moreover, the widely employed analytical assay, namely the enzyme-linked immunosorbent assay (ELISA), has shown that therapy with SST before subjection to LPS markedly de-escalated the formation of inflammatory mediators, for instance, prostaglandin E2, TNF-α, and IL-1β. In addition, immunoblotting inspection has demonstrated that the introduction of SST before LPS exposure culminated in the significant reduction in the LPS-prompted expression of the trio, namely NF-κB p-p65, inducible NO synthase, and cyclooxygenase-2 [37]. These outcomes depicted that SST has the aptitude to inhibit the stimulation of microglia and the NF-κB mechanism, and, consequently, declines the nerve cell inflammation and oxidative destruction, and finally suppresses the DArgic nerve cell deprivation elicited by LPS exposure in nerve cell deteriorating diseases such as PD [29]. Howbeit, more investigation is greatly needed in this particular domain to decipher the restorative aptitude of SST in PD.

**Table 1:** The amino acid units and amino acid sequence of fundamental neuropeptides that contribute to significant neuroprotection in Parkinson’s disease.

Neuropeptide	Amino Acid Units	Amino Acid Sequence	Ref.
Substance P	11	H-Arg-Pro-Lys-Pro-Gln-Gln-Phe-Ple-Gly-Leu-Met-NH_2_	[111,112]
Ghrelin	28	NH_2_-Gly-Ser-[Ser(n-octanoyl)]-Phe-Leu-Ser-Pro-Glu-His-Gln-Arg-Val-Gln-Gln-Arg-Lys-Glu-Ser-Lys-Lys-Pro-Pro-Ala-Lys-Leu-Gln-Pro-Arg-COOH	[131]
Neuropeptide Y	36	Tyr-Pro-Ser-Lys-Pro-Asp-Asn-Pro-Gly-Glu-Asp-Ala-Pro-Ala-Glu-Asp-Leu-Ala-Arg-Tyr-Tyr-Ser-Ala-Leu-Arg-His-Tyr-Ile-Asn-Leu-Ile-Thr-Arg-Gln-Arg-Tyr-NH_2_	[156]
Neurotensin	13	pyr-Glu-Leu-Tyr-Glu-Asn-Lys-Pro-Arg-Arg-Pro-Tyr-Ile-Leu-OH	[177]
Pituitary adenylate cyclase-activating polypeptide	27	H-His-Ser-Asp-Gly-Ile-Phe-Thr-Asp-Ser-Tyr-Ser-Arg-Tyr-Arg-Lys-Gln-Met-Ala-Val-Lys-Lys-Tyr-Leu-Ala-Ala-Val-Leu-NH_2_	[193,194]
	38	H-His-Ser-Asp-Gly-Ile-Phe-Thr-Asp-Ser-Tyr-Ser-Arg-Tyr-Arg-Lys-Gln-Met-Ala-Val-Lys-Lys-Tyr-Leu-Ala-Ala-Val-Leu-Gly-Lys-Arg-Tyr-Lys-Gln-Arg-Val-Lys-Asn-Lys-NH_2_	
Nesfatin-1	82	Val-Pro-Ile-Asp-Ile-Asp-Lys-Thr-Lys-Val-Gln-Asn-Ile-His-Pro-Val-Glu-Ser-Ala-Lys-Ile-Glu-Pro-Pro-Asp-Thr-Gly-Leu-Tyr-Tyr-Asp-Glu-Tyr-Leu-Lys-Gln-Val-Ile-Asp-Val-Leu-Glu-Thr-Asp-Lys-His-Phe-Arg-Glu-Lys-Leu-Gln-Lys-Ala-Asp-Ile-Glu-Glu-Ile-Lys-Ser-Gly-Arg-Leu-Ser-Lys-Glu-Leu-Asp-Leu-Val-Ser-His-His-Val-Arg-Thr-Lys-Leu-Asp-Glu-Leu	[218]
Somatostatin	14	Ala-Gly-Cys-Lys-Asn-Phe-Phe-Trp-Lys-Thr-Phe-Thr-Ser-Cys	[229]
	28	Ser-Ala-Asn-Ser-Asn-Pro-Ala-Met-Ala-Pro-Arg-Glu-Arg-Lys-Ala-Gly-Cys-Lys-Asn-Phe-Phe-Trp-Lys-Thr-Phe-Thr-Ser-Cys	

## 4. Conclusions

PD, a multifaceted and incapacitating condition, is depicted by the devastation of DA-forming nerve cells inside the SN-PC, which ultimately culminates into DA scantiness in the striatal area. The malady is represented by four key manifestations, viz., rigor, postural deformities, tremor, and bradykinesia. Although the exact etiopathology of the condition is perplexing, multifactorial, and equivocal, the procured data greatly suggested that aging, genetic predisposition, and subjection to environmental toxins unitedly participate in the emergence of PD. The pathological pathways indulged in PD encompasses the clumping of α-synuclein within the LBs and lewy neurites, oxidative stress, apoptosis, neuronal-inflammation, and abnormalities in the operation of mitochondria, ALP, and UPS. Presently, the therapy with the help of DA precursor (levodopa), DA agonists, COMT inhibitors, and MAO-B inhibitors fundamentally concentrates on the mitigation of disease-concerned manifestations, but hitherto no therapeutic approach has been signified to halt the advancement of the disease. NPs are termed as tiny, protein-comprehending additional messenger substances that are fundamentally generated and liberated by nerve cells inside the entire nervous system. NPs are synthesized in the cell body from their large protein precursors, designated as prepropeptides (which are synthesized on palade granules at the endoplasmic reticulum and processed by means of the Golgi apparatus). The NPs are mainly transcripted and translated from the prepropeptides genes. Additionally, the activation of prepropeptides carried by proteases results in the conversion of prepropeptides into propeptides, and lastly, NPs are derived, following the stimulation of converting biocatalysts. Proteolytic processing consequentially partakes in the activation, partial inactivation, or inactivation of the modulatory peptides, such as NPs. Proteases bring about the breakdown, and as a result, may activate, inactivate, or liberate other proteins/peptides. Amongst the two, i.e., cell-surface proteases and proteases, which are liberated by the cells, the cell-surface proteases exhibit enormously greater regulatory and specialized functions. The cell-surface proteases display their action by deteriorating the two, i.e., the bioactive peptides and the cellular functions. Aside from the proteolytic deterioration and synthetic alterations, the removal by means of filtration/diffusion is of utmost and remarkable importance. The preponderance of the cell-surface proteases has been originally depicted as the clearing of biocatalysts, even though they exhibit the potential to cleave the peptides possessing not more than 80 residues. To date, they are presumed as the modulatory proteases (such as ACE, ECE, NEP, and DPP IV) that possess the tremendous tendency to modulate the activation or inactivation of NPs. After the completion of the synthesis, NPs are stored within the large and dense vesicles, and finally they are released via exocytosis. Subsequently, NPs undergo interaction with GPCRs so as to instigate their biological actions and regulate nerve cell operation. NPs consequentially participate in the regulation of the immune system, biological equilibrium (such as the biotransformation of blood sugar, blood pressure, equilibrium in the water content, stress reaction, feeding behavior, and pain), and nerve cell protection.

In the current methodical review, the authors spotlighted the emerging status and nerve cell protective role of various NPs, encompassing SP, ghrelin, NPY, neurotensin, PACAP, nesfatin-1, and SST in PD. Pursuant to present-day literature, changes in the expressions of above-stated NPs, as well as their respective GPCRs, were significantly observed within the PD-associated areas, predominantly the SN-striatal area. Numerous pathways, comprehending the suppression of microglia stimulation, cytotoxicity, programmed cell death, oxidative stress, inflammation, autophagy, nerve cell toxicity, the stimulation of chondriosomal bioenergetics, and the de-escalation of disease-related manifestations, emerge to be entailed in NPs-prompted nerve cell protective action in PD. In addition, the trio, namely analogs (septide, Dpr3ghr, HM01, neurotensin2, and neurotensin4), agonists (septide, senktide, HM01, and PACAP (1–38)), and antagonists (NAT, L-733060, LY303870, and [D-Lys3]-GHRP6), of these NPs were as well employed in order to safeguard against nerve cell toxin-precipitated DArgic nerve cell devastation, as well as motor and nonmotor deficiencies, thereby furnishing a newfangled and propitious therapeutic perspective for the management of PD.

At present, there are multifarious concerns associated with the peptide therapy, such as the natural peptides exhibiting deprived absorption, distribution, metabolism, and excretion (ADME) profiles, together with little half-life, expeditious clearance, and little solubility/permeability. These issues might be addressed by the continual exploration in this respective domain so as to discover whether these alterations in the content of NPs within the plasma/CSF can be employed as biomarkers in PD. Numerous strategies have emerged with the potential to enormously upgrade the peptide stability by means of structural alteration, escalating permeability and half-life, and de-escalating the clearance and proteolysis. Multiple strategies not only upgrade the stability, but as well upgrade the additional ADME properties; for instance, the conjugation to larger molecules might upgrade the stability and plummet the renal clearance, and cyclic ring formation might elevate the stability as well as the permeability. Further explorations of pharmacokinetic and pharmacodynamic properties/models might render detailed insights in the area of peptide therapy evolution with tremendous efficacy and safety, and minimal deleterious repercussions [235].

In addition, further experimental and clinical investigations are remarkably required in order to attain an exhaustive knowledge regarding NPs, their analogs, agonists, antagonists, their mode of action in bringing nerve cell protective action and overcoming nerve cell devastation, and to open neoteric, exciting, and magnificent gateways in the therapy of PD.

## Abbreviations

AC, adenyl cyclase; ACE, angiotensin-converting enzyme; ACh, acetylcholine; *ADCYAP1*, *adenylate cyclase activating polypeptide 1*; ADME, absorption, distribution, metabolism, and excretion; ALP, autophagy lysosomal pathway; AMPK, 5′ adenosine monophosphate-activated protein kinase; ATP13A2, neuronal P-type adenosine triphosphate (ATP)ase gene; BAs, biogenic amines; Bax, Bcl-2-associated X protein; Bcl-2, B-cell lymphoma-2; BDNF, brain-derived neurotrophic factor; BiP, binding immunoglobulin protein; BNST, bed nucleus of the stria terminalis; C, carboxy; Ca^2+^, calcium ions; cAMP, cyclic adenosine monophosphate; CArgic, catecholaminergic; CAT, catalase; CCl_4_, carbon tetrachloride; CD11b, cluster of differentiation molecule 11b; CH_3_OH, methanol; CNS, central nervous system; C=O, carbonyl; COMT, catechol-O-methyltransferase; CR, caloric restriction; CSF, cerebrospinal fluid; *CTSB*, *cathepsin B*; *CTSD*, *cathepsin D*; Cu, copper; Cu-Zn SOD, copper-zincsuperoxide dismutase; C57BL/6, C57 black 6; DA, dopamine; DArgic, dopaminergic; DJ-1, protein deglycase; DNA, deoxyribonucleic acid; DPP IV, dipeptidyl peptidase IV; D2R, DA 2 receptors; ECE, endothelin-converting enzyme; ELISA, enzyme-linked immunosorbent assay; ENS, enteric nervous system; ER stress, endoplasmic reticulum stress; ERK, extracellular signal-regulated kinase; ETC, electron transport chain; Fe, iron; *GBA*, glucocerebrosidase; GH, growth hormone; GHIH, growth hormone-inhibiting hormone; GhrelinR, ghrelin receptor; GHS-R1a, growth hormone secretagogue receptor 1a; G_i_, G inhibitory; GPCRs, G-protein coupled receptors; GRP78, 78-kDa glucose-regulated protein; GSH, glutathione; HCN, hyperpolarization-activated cyclic nucleotide-gated channels; HSC70, heat shock cognate protein 70;HSPs, heat shock proteins; HSP35, hereditary spastic paraplegia type 35; HTRA2, high-temperature requirement A2; H_2_O, water; IFNs, interferons; IFN-γ, interferon-gamma; ILs, interleukins; IL-1β, interleukin-1β; IL-6, interleukin-6; INS, intrinsic nervous system; InsP_3_, inositol 1,4,5-trisphosphate; K^+^, potassium; KATP, ATP-sensitive potassium channel; LAMP1, lysosome-associated membrane protein 1; LAMP2A, lysosome-associated membrane protein 2A; LBs, lewy bodies; LC, locus coeruleus; LC3-II, microtubule-associated protein light chain 3-phosphatidylethanolamine conjugate; LDH, lactate dehydrogenase; LOOH, lipid hydroperoxides; LPS, lipopolysaccharide; *LRRK2*, *leucine-rich repeat kinase 2*; LY303870, lanepitant; MAO-B, monoamine oxidase B; MAPK, mitogen-activated protein kinase; MDA, malondialdehyde; MES23.5, DArgic nerve cell line; MGO, methylglyoxal; MMP-9, matrix metallopeptidase 9; MPP^+^, 1-methyl-4-phenylpyridinium ion; MPTP, 1-methyl-4-phenyl-1,2,3,6-tetrahydropyridine; mRNA, messenger ribonucleic acid; MtMP, mitochondrial membrane potential; NADPH, reduced nicotinamide adenine dinucleotide phosphate; NAT, N-acetyl-L-tryptophan; NEP, neutral endopeptidase; NF-κB, Nuclear Factor kappa-light-chain-enhancer of activated B cells; NK, neurokinin; NKA, neurokinin A; NKB, neurokinin B; NK1, neurokinin 1; NK2, neurokinin 2; NK3, neurokinin 3; NK1R, NK1 receptor; NMDA, N-methyl-D-aspartate; NO, nitric oxide; NPs, neuropeptides; NPY, neuropeptide Y; NTR1, neurotensin receptor 1; NTR2, neurotensin receptor 2; NTR3, neurotensin receptor 3; NUCB2, nucleobindin-2; O_2_, oxygen; PA28, proteasome activator 28;PA700, proteasome activator 700; PAC1, PACAP type I receptor; PACAP, pituitary adenylate cyclase-activating polypeptide; *Parkin*, *Parkin RBR E3 ubiquitin–protein ligase*; PCNA, proliferating cell nuclear antigen; p-CREB, phosphorylated cAMP-response element binding protein; PD, Parkinson’s disease; PERC, perchloroethylene; PFF, preformed fibrils; PGCs, progenitor cells; PI3K, phosphoinositide 3-kinase; *PINK1*, *PTEN-induced kinase 1*; PKA, protein kinase A; PKB/Akt, protein kinase B signaling pathway; PKCδ, protein kinase C delta; PLC-β, phospholipase C-beta; PNS, peripheral nervous system; PP, pancreatic polypeptide; PYY, peptide YY; RGCs, retinal ganglion cells; ROS, reactive oxygen species; S, sulphur; SH-SY5Y, human neuroblastoma cells; *SMPD1*, *sphingomyelin phosphodiesterase 1*,SN, substantia nigra; *SNCA*, α-synuclein; SN-PC, substantia nigra pars compacta; SOD, superoxide dismutase; SP, substance P; SST, somatostatin; SSTR1, SST receptor 1; SSTR2, SST receptor 2; SSTR3, SST receptor 3; SSTR4, SST receptor 4; SSTR5, SST receptor 5; SVZ, subventricular zone; SYNJ1, synaptojanin 1; TAC1, tachykinin precursor 1 gene; TCE, trichloroethylene; TH, tyrosine hydroxylase; TK, tachykinin; *TMEM175*, *transmembrane protein 175*; TNF-α, tumor necrosis factor-alpha; TRAF6, tumor necrosis factor receptor-associated factor 6; UCHL1, *ubiquitin carboxy* (C)*-terminal hydrolase L1*; UCP2, uncoupling protein 2; UPS, ubiquitin-proteasome system; VIP, vasoactive intestinal polypeptide; VPAC1, VIP receptor 1; VPAC2, VIP receptor 2; VPS10P, vacuolar protein sorting 10 protein; VPS35, vacuolar protein sorting 35; XBP1s, spliced form of X-box binding protein 1; Y_1_R, Y_1_ receptor; Y_2_R, Y_2_ receptor; Y_3_R, Y_3_ receptor; Y_4_R, Y_4_ receptor; Y_5_R, Y_5_ receptor; Y_6_R, Y_6_ receptor; Zn, zinc; 5-HT, 5-hydroxytryptamine; 6-OHDA, 6-hydroxy DA; 7TM, seven transmembrane domain.

## References

[1] Palacios-Sánchez L., Nupan M.T., Botero-Meneses J.S. (2017). James Parkinson and His Essay on “Shaking Palsy”, Two Hundred Years Later. Arq. Neuro-Psiquiatr..

[2] Hayes M.T. (2019). Parkinson’s Disease and Parkinsonism. Am. J. Med..

[3] Dawson T.M. (2000). Parkinson’s Disease: Clinical Manifestations and Treatment. Int. Rev. Psychiatry.

[4] Surmeier D.J. (2018). Determinants of Dopaminergic Neuron Loss in Parkinson’s Disease. FEBS J..

[5] Muangpaisan W., Hori H., Brayne C. (2009). Systematic Review of the Prevalence and Incidence of Parkinson’s Disease in Asia. J. Epidemiol..

[6] Zou Y.-M., Liu J., Tian Z.-Y., Lu D., Zhou Y.-Y. (2015). Systematic Review of the Prevalence and Incidence of Parkinson’s Disease in the People’s Republic of China. Neuropsychiatr. Dis. Treat..

[7] Picillo M., Nicoletti A., Fetoni V., Garavaglia B., Barone P., Pellecchia M.T. (2017). The Relevance of Gender in Parkinson’s Disease: A Review. J. Neurol..

[8] Pang S.Y.Y., Ho P.W.L., Liu H.F., Leung C.T., Li L., Chang E.E.S., Ramsden D.B., Ho S.L. (2019). The Interplay of Aging, Genetics and Environmental Factors in the Pathogenesis of Parkinson’s Disease. Transl. Neurodegener..

[9] Veldman B.A.J., Wijn A.M., Knoers N., Praamstra P., Horstink M.W.I.M. (1998). Genetic and Environmental Risk Factors in Parkinson’s Disease. Clin. Neurol. Neurosurg..

[10] Georgiou A., Demetriou C.A., Christou Y.P., Heraclides A., Leonidou E., Loukaides P., Yiasoumi E., Pantziaris M., Kleopa K.A., Papacostas S.S. (2019). Genetic and Environmental Factors Contributing to Parkinson’s Disease: A Case-Control Study in the Cypriot Population. Front. Neurol..

[11] Wang H., Yang F., Zhang S., Xin R., Sun Y. (2021). Genetic and Environmental Factors in Alzheimer’s and Parkinson’s Diseases and Promising Therapeutic Intervention via Fecal Microbiota Transplantation. NPJ Park. Dis..

[12] Thakur M., Rachamalla M., Niyogi S., Datusalia A.K., Flora S.J.S. (2021). Molecular Mechanism of Arsenic-Induced Neurotoxicity including Neuronal Dysfunctions. Int. J. Mol. Sci..

[13] Patel R.S., Rachamalla M., Chary N.R., Shera F.Y., Tikoo K., Jena G. (2012). Cytarabine Induced Cerebellar Neuronal Damage in Juvenile Rat: Correlating Neurobehavioral Performance with Cellular and Genetic Alterations. Toxicology.

[14] Bhardwaj S., Kesari K.K., Rachamalla M., Mani S., Ashraf G.M., Jha S.K., Kumar P., Ambasta R.K., Dureja H., Devkota H.P. (2021). CRISPR/Cas9 Gene Editing: New Hope for Alzheimer’s Disease Therapeutics. J. Adv. Res..

[15] Kouli A., Torsney K.M., Kuan W.-L. (2018). Parkinson’s Disease: Etiology, Neuropathology, and Pathogenesis. Parkinson’s Disease: Pathogenesis and Clinical Aspects.

[16] Iarkov A., Barreto G.E., Grizzell J.A., Echeverria V. (2020). Strategies for the Treatment of Parkinson’s Disease: Beyond Dopamine. Front. Aging Neurosci..

[17] Armstrong M.J., Okun M.S. (2020). Diagnosis and Treatment of Parkinson Disease: A Review. JAMA—J. Am. Med. Assoc..

[18] Kakkar A.K., Dahiya N. (2015). Management of Parkinsonߣs Disease: Current and Future Pharmacotherapy. Eur. J. Pharmacol..

[19] Carniglia L., Ramírez D., Durand D., Saba J., Turati J., Caruso C., Scimonelli T.N., Lasaga M. (2017). Neuropeptides and Microglial Activation in Inflammation, Pain, and Neurodegenerative Diseases. Mediat. Inflamm..

[20] Hökfelt T., Barde S., Xu Z.-Q.D., Kuteeva E., Rüegg J., Le Maitre E., Risling M., Kehr J., Ihnatko R., Theodorsson E. (2018). Neuropeptide and Small Transmitter Coexistence: Fundamental Studies and Relevance to Mental Illness. Front. Neural Circuits.

[21] Merighi A. (2002). Costorage and Coexistence of Neuropeptides in the Mammalian CNS. Prog. Neurobiol..

[22] Merighi A., Salio C., Ferrini F., Lossi L. (2011). Neuromodulatory Function of Neuropeptides in the Normal CNS. J. Chem. Neuroanat..

[23] Surget A., Leman S., Griebel G., Belzung C., Yalcin I. (2006). Neuropeptides in Psychiatric Diseases: An Overview with a Particular Focus on Depression and Anxiety Disorders. CNS Neurol. Disord.—Drug Targets.

[24] Barrett A.J. (2000). Proteases. Curr. Protoc. Protein Sci..

[25] Mentlein R. (2004). Cell-Surface Peptidases. Int. Rev. Cytol..

[26] Zhang Y., Wang Z., Parks G.S., Civelli O. (2012). Novel Neuropeptides as Ligands of Orphan G Protein-Coupled Receptors. Curr. Pharm. Des..

[27] Hökfelt T., Bartfai T., Bloom F. (2003). Neuropeptides: Opportunities for Drug Discovery. Lancet Neurol..

[28] Palkovits M. (1995). Neuropeptide Messenger Plasticity in the CNS Neurons Following Axotomy. Mol. Neurobiol..

[29] Catalani E., De Palma C., Perrotta C., Cervia D. (2017). Current Evidence for a Role of Neuropeptides in the Regulation of Autophagy. BioMed Res. Int..

[30] Wang S.-Y., Chen L., Xue Y., Xia Y.-J. (2012). Substance P Prevents 1-methyl-4-Phenylpyridiniuminduced Cytotoxicity through Inhibition of Apoptosis via Neurokinin-1 Receptors in MES23.5 Cells. Mol. Med. Rep..

[31] Shi L., Du X., Jiang H., Xie J. (2017). Ghrelin and Neurodegenerative Disorders—A Review. Mol. Neurobiol..

[32] Bayliss J.A., Lemus M., Santos V.V., Deo M., Elsworth J.D., Andrews Z.B. (2016). Acylated but not Des-acyl Ghrelin is Neuroprotective in an MPTP Mouse Model of Parkinson’s Disease. J. Neurochem..

[33] Li C., Wu X., Liu S., Zhao Y., Zhu J., Liu K. (2019). Roles of Neuropeptide Y in Neurodegenerative and Neuroimmune Diseases. Front. Neurosci..

[34] Lazarova M., Popatanasov A., Klissurov R., Stoeva S., Pajpanova T., Kalfin R., Tancheva L. (2018). Preventive Effect of Two New Neurotensin Analogues on Parkinson’s Disease Rat Model. J. Mol. Neurosci..

[35] Maasz G., Zrinyi Z., Reglodi D., Petrovics D., Rivnyak A., Kiss T., Jungling A., Tamas A., Pirger Z. (2017). Pituitary Adenylate Cyclase-Activating Polypeptide (PACAP) Has Neuroprotective Function in Dopamine-Based Neurodegeneration Developed in Two Parkinsonian Models. Dis. Model. Mech..

[36] Dong D., Xie J., Wang J. (2019). Neuroprotective Effects of Brain-Gut Peptides: A Potential Therapy for Parkinson’s Disease. Neurosci. Bull..

[37] Bai L., Zhang X., Li X., Liu N., Lou F., Ma H., Luo X., Ren Y. (2015). Somatostatin Prevents Lipopolysaccharide-Induced Neurodegeneration in the Rat Substantia Nigra by Inhibiting the Activation of Microglia. Mol. Med. Rep..

[38] Srinivasan E., Chandrasekhar G., Chandrasekhar P., Anbarasu K., Vickram A.S., Karunakaran R., Rajasekaran R., Srikumar P.S. (2021). Alpha-Synuclein Aggregation in Parkinson’s Disease. Front. Med..

[39] Coelho M., Ferreira J., Rosa M., Sampaio C. (2008). Treatment Options for Non-motor Symptoms in Late-Stage Parkinson’s Disease. Expert Opin. Pharmacother..

[40] Yang W., Hamilton J.L., Kopil C., Beck J.C., Tanner C.M., Albin R.L., Dorsey E.R., Dahodwala N., Cintina I., Hogan P. (2020). Current and Projected Future Economic Burden of Parkinson’s Disease in the U.S. NPJ Park. Dis..

[41] Cacabelos R. (2017). Parkinson’s Disease: From Pathogenesis to Pharmacogenomics. Int. J. Mol. Sci..

[42] Hirsch L., Jette N., Frolkis A., Steeves T., Pringsheim T. (2016). The Incidence of Parkinson’s Disease: A Systematic Review and Meta-Analysis. Neuroepidemiology.

[43] Driver J.A., Logroscino G., Gaziano J.M., Kurth T. (2009). Incidence and Remaining Lifetime Risk of Parkinson Disease in Advanced Age. Neurology.

[44] Crispino P., Gino M., Barbagelata E., Ciarambino T., Politi C., Ambrosino I., Ragusa R., Marranzano M., Biondi A., Vacante M. (2021). Gender Differences and Quality of Life in Parkinson’s Disease. Int. J. Environ. Res. Public Health.

[45] Cerri S., Mus L., Blandini F. (2019). Parkinson’s Disease in Women and Men: What’s the Difference?. J. Parkinson’s Dis..

[46] Maiti P., Manna J., Dunbar G.L. (2017). Current Understanding of the Molecular Mechanisms in Parkinson’s Disease: Targets for Potential Treatments. Transl. Neurodegener..

[47] Liu H., Koros C., Strohäker T., Schulte C., Bozi M., Varvaresos S., Ibáñez de Opakua A., Simitsi A.M., Bougea A., Voumvourakis K. (2021). A Novel SNCA A30G Mutation Causes Familial Parkinson’s Disease. Mov. Disord..

[48] Magistrelli L., Contaldi E., Comi C. (2021). The Impact of *SNCA* Variations and Its Product Alpha-Synuclein on Non-Motor Features of Parkinson’s Disease. Life.

[49] Kamienieva I., Duszyński J., Szczepanowska J. (2021). Multitasking Guardian of Mitochondrial Quality: Parkin Function and Parkinson’s Disease. Transl. Neurodegener..

[50] Nawaz M.S., Asghar R., Pervaiz N., Ali S., Hussain I., Xing P., Bao Y., Abbasi A.A. (2020). Molecular Evolutionary and Structural Analysis of Human UCHL1 Gene Demonstrates the Relevant Role of Intragenic Epistasis in Parkinson’s Disease and Other Neurological Disorders. BMC Evol. Biol..

[51] Vizziello M., Borellini L., Franco G., Ardolino G. (2021). Disruption of Mitochondrial Homeostasis: The Role of PINK1 in Parkinson’s Disease. Cells.

[52] Repici M., Giorgini F. (2019). DJ-1 in Parkinson’s Disease: Clinical Insights and Therapeutic Perspectives. J. Clin. Med..

[53] Hur E.-M., Lee B.D. (2021). LRRK2 at the Crossroad of Aging and Parkinson’s Disease. Genes.

[54] Cerri S., Ghezzi C., Ongari G., Croce S., Avenali M., Zangaglia R., Di Monte D.A., Valente E.M., Blandini F. (2021). GBA Mutations Influence the Release and Pathological Effects of Small Extracellular Vesicles from Fibroblasts of Patients with Parkinson’s Disease. Int. J. Mol. Sci..

[55] Luo A., Xu Z., Liao S. (2021). VPS35, the Core Component of the Retromer Complex, and Parkinson’s Disease. Ibrain.

[56] Park J.-S., Blair N.F., Sue C.M. (2015). The Role of ATP13A2 in Parkinson’s Disease: Clinical Phenotypes and Molecular Mechanisms. Mov. Disord..

[57] He Y.-C., Huang P., Li Q.-Q., Sun Q., Li D.-H., Wang T., Shen J.-Y., Du J.-J., Cui S.-S., Gao C. (2017). Mutation Analysis ofHTRA2Gene in Chinese Familial Essential Tremor and Familial Parkinson’s Disease. Park. Dis..

[58] Lesage S., Mangone G., Tesson C., Bertrand H., Benmahdjoub M., Kesraoui S., Arezki M., Singleton A., Corvol J.-C., Brice A. (2021). Clinical Variability of SYNJ1-Associated Early-Onset Parkinsonism. Front. Neurol..

[59] Behl T., Madaan P., Sehgal A., Singh S., Sharma N., Bhatia S., Al-Harrasi A., Chigurupati S., Alrashdi I., Bungau S.G. (2021). Elucidating the Neuroprotective Role of PPARs in Parkinson’s Disease: A Neoteric and Prospective Target. Int. J. Mol. Sci..

[60] Thirugnanam T., Santhakumar K. (2022). Chemically Induced Models of Parkinson’s Disease. Comp. Biochem. Physiol. Part C Toxicol. Pharmacol..

[61] Kochmanski J., VanOeveren S.E., Patterson J.R., Bernstein A.I. (2019). Developmental Dieldrin Exposure Alters DNA Methylation at Genes Related to Dopaminergic Neuron Development and Parkinson’s Disease in Mouse Midbrain. Toxicol. Sci..

[62] Jia Z., Misra H.P. (2007). Exposure to Mixtures of Endosulfan and Zineb Induces Apoptotic and Necrotic Cell Death in SH-SY5Y Neuroblastoma Cells, In Vitro. J. Appl. Toxicol..

[63] Martin C.A., Myers K.M., Chen A., Martin N.T., Barajas A., Schweizer F.E., Krantz D.E. (2016). Ziram, a Pesticide Associated with Increased Risk for Parkinson’s Disease, Differentially Affects the Presynaptic Function of Aminergic and Glutamatergic Nerve Terminals at the Drosophila Neuromuscular Junction. Exp. Neurol..

[64] Pouchieu C., Piel C., Carles C., Gruber A., Helmer C., Tual S., Marcotullio E., LeBailly P., Baldi I. (2018). Pesticide Use in Agriculture and Parkinson’s Disease in the AGRICAN Cohort Study. Int. J. Epidemiol..

[65] Anderson C.C., Marentette J.O., Rauniyar A.K., Prutton K.M., Khatri M., Matheson C., Reisz J.A., Reigan P., D’Alessandro A., Roede J.R. (2021). Maneb Alters Central Carbon Metabolism and Thiol Redox Status in a Toxicant Model of Parkinson’s Disease. Free Radic. Biol. Med..

[66] Lock E.A., Zhang J., Checkoway H. (2013). Solvents and Parkinson Disease: A Systematic Review of Toxicological and Epidemiological Evidence. Toxicol. Appl. Pharmacol..

[67] Goldman S.M., Quinlan P.J., Ross G.W., Marras C., Meng C., Bhudhikanok G.S., Comyns K., Korell M., Chade A.R., Kasten M. (2012). Solvent Exposures and Parkinson Disease Risk in Twins. Ann. Neurol..

[68] Behl T., Madaan P., Sehgal A., Singh S., Anwer K., Makeen H.A., Albratty M., Mohan S., Bungau S. (2022). Mechanistic Insights Expatiating the Redox-Active-Metal-Mediated Neuronal Degeneration in Parkinson’s Disease. Int. J. Mol. Sci..

[69] Wang X., Michaelis E.K. (2010). Selective Neuronal Vulnerability to Oxidative Stress in the Brain. Front. Aging Neurosci..

[70] Parker W.D., Parks J.K., Swerdlow R.H. (2008). Complex I Deficiency in Parkinson’s Disease Frontal Cortex. Brain Res..

[71] Anderson R.F., Harris T.A. (2003). Dopamine and Uric Acid Act as Antioxidants in the Repair of DNA Radicals: Implications in Parkinson’s Disease. Free Radic. Res..

[72] Lotharius J., Brundin P. (2002). Pathogenesis of Parkinson’s Disease: Dopamine, Vesicles and α-synuclein. Nat. Rev. Neurosci..

[73] Aoyama K. (2021). Glutathione in the Brain. Int. J. Mol. Sci..

[74] Wei Z., Li X., Li X., Liu Q., Cheng Y. (2018). Oxidative Stress in Parkinson’s Disease: A Systematic Review and Meta-Analysis. Front. Mol. Neurosci..

[75] Wang J.-Y., Zhuang Q.-Q., Zhu L.-B., Zhu H., Li T., Li R., Chen S.-F., Huang C.-P., Zhang X., Zhu J.-H. (2016). Meta-Analysis of Brain Iron Levels of Parkinson’s Disease Patients Determined by Postmortem and MRI Measurements. Sci. Rep..

[76] De Farias C.C., Maes M., Bonifácio K.L., Bortolasci C.C., de Souza Nogueira A., Brinholi F.F., Matsumoto A.K., do Nascimento M.A., De Melo L.B., Nixdorf S.L. (2016). Highly Specific Changes in Antioxidant Levels and Lipid Peroxidation in Parkinson’s Disease and Its Progression: Disease and Staging Biomarkers and New Drug Targets. Neurosci. Lett..

[77] Jiménez-Jiménez F., Alonso-Navarro H., Herrero M., García-Martín E., Agúndez J. (2016). An Update on the Role of Nitric Oxide in the Neurodegenerative Processes of Parkinson’s Disease. Curr. Med. Chem..

[78] Subedi L., Gaire B., Parveen A., Kim S.-Y. (2021). Nitric Oxide as a Target for Phytochemicals in Anti-Neuroinflammatory Prevention Therapy. Int. J. Mol. Sci..

[79] Senkevich K., Gan-Or Z. (2020). Autophagy Lysosomal Pathway Dysfunction in Parkinson’s Disease; Evidence from Human Genetics. Park. Relat. Disord..

[80] Alvarez-Erviti L., Rodriguez-Oroz M.C., Cooper J.M., Caballero C., Ferrer I., Obeso J.A., Schapira A.H.V. (2010). Chaperone-Mediated Autophagy Markers in Parkinson Disease Brains. Arch. Neurol..

[81] Hou X., Watzlawik J.O., Fiesel F.C., Springer W. (2020). Autophagy in Parkinson’s Disease. J. Mol. Biol..

[82] Lynch-Day M.A., Mao K., Wang K., Zhao M., Klionsky D.J. (2012). The Role of Autophagy in Parkinson’s Disease. Cold Spring Harb. Perspect. Med..

[83] Beilina A., Cookson M.R. (2016). Genes Associated with Parkinson’s Disease: Regulation of Autophagy and Beyond. J. Neurochem..

[84] McNaught K.S., Jenner P. (2001). Proteasomal Function Is Impaired in Substantia Nigra in Parkinson’s Disease. Neurosci. Lett..

[85] Zheng Q., Huang T., Zhang L., Zhou Y., Luo H., Xu H., Wang X. (2016). Dysregulation of Ubiquitin-Proteasome System in Neurodegenerative Diseases. Front. Aging Neurosci..

[86] Dawson T.M., Dawson V.L. (2003). Rare Genetic Mutations Shed Light on the Pathogenesis of Parkinson Disease. J. Clin. Investig..

[87] Zucchelli S., Codrich M., Marcuzzi F., Pinto M., Vilotti S., Biagioli M., Ferrer I., Gustincich S. (2010). TRAF6 Promotes Atypical Ubiquitination of Mutant DJ-1 and Alpha-Synuclein and Is Localized to Lewy Bodies in Sporadic Parkinson’s Disease Brains. Hum. Mol. Genet..

[88] McNaught K.S.P., Belizaire R., Isacson O., Jenner P., Olanow C.W. (2003). Altered Proteasomal Function in Sporadic Parkinson’s Disease. Exp. Neurol..

[89] Prasuhn J., Davis R.L., Kumar K.R. (2021). Targeting Mitochondrial Impairment in Parkinson’s Disease: Challenges and Opportunities. Front. Cell Dev. Biol..

[90] Park J.-S., Davis R.L., Sue C.M. (2018). Mitochondrial Dysfunction in Parkinson’s Disease: New Mechanistic Insights and Therapeutic Perspectives. Curr. Neurol. Neurosci. Rep..

[91] Bose A., Beal M.F. (2016). Mitochondrial Dysfunction in Parkinson’s Disease. J. Neurochem..

[92] Tanner C.M., Kamel F., Ross G.W., Hoppin J.A., Goldman S.M., Korell M., Marras C., Bhudhikanok G.S., Kasten M., Chade A.R. (2011). Rotenone, Paraquat, and Parkinson’s Disease. Environ. Health Perspect..

[93] Bogaerts V., Theuns J., Van Broeckhoven C. (2008). Genetic Findings in Parkinson’s Disease and Translation into Treatment: A Leading Role for Mitochondria?. Genes Brain Behav..

[94] Anglade P., Vyas S., Javoy-Agid F., Herrero M.T., Michel P.P., Marquez J., Mouatt-Prigent A., Ruberg M., Hirsch E.C., Agid Y. (1997). Apoptosis and Autophagy in Nigral Neurons of Patients with Parkinson’s Disease. Histol. Histopathol..

[95] Hunot S., Brugg B., Ricard D., Michel P.P., Muriel M.-P., Ruberg M., Faucheux B.A., Agid Y., Hirsch E.C. (1997). Nuclear Translocation of NF-κB is Increased in Dopaminergic Neurons of Patients with Parkinson Disease. Proc. Natl. Acad. Sci. USA.

[96] Moujalled D., Strasser A., Liddell J.R. (2021). Molecular Mechanisms of Cell Death in Neurological Diseases. Cell Death Differ..

[97] Mount M.P., Lira A., Grimes D., Smith P.D., Faucher S., Slack R., Anisman H., Hayley S., Park D.S. (2007). Involvement of Interferon-γ in Microglial-Mediated Loss of Dopaminergic Neurons. J. Neurosci..

[98] Bellucci A., Bubacco L., Longhena F., Parrella E., Faustini G., Porrini V., Bono F., Missale C., Pizzi M. (2020). Nuclear Factor-κB Dysregulation and α-Synuclein Pathology: Critical Interplay in the Pathogenesis of Parkinson’s Disease. Front. Aging Neurosci..

[99] Hartmann A., Mouatt-Prigent A., Vila M., Abbas N., Perier C., Faucheux B.A., Vyasb S., Hirsch E.C. (2002). Increased Expression and Redistribution of the Antiapoptotic Molecule Bcl-xL in Parkinson’s Disease. Neurobiol. Dis..

[100] Badanjak K., Fixemer S., Smajić S., Skupin A., Grünewald A. (2021). The Contribution of Microglia to Neuroinflammation in Parkinson’s Disease. Int. J. Mol. Sci..

[101] Zhang W., Wang T., Pei Z., Miller D.S., Wu X., Block M.L., Wilson B., Zhang W., Zhou Y., Hong J.-S. (2005). Aggregated α-synuclein Activates Microglia: A Process Leading to Disease Progression in Parkinson’s Disease. FASEB J..

[102] He S., Zhong S., Liu G., Yang J. (2021). Alpha-Synuclein: The Interplay of Pathology, Neuroinflammation, and Environmental Factors in Parkinson’s Disease. Neurodegener. Dis..

[103] Abeliovich A., Schmitz Y., Fariñas I., Choi-Lundberg D., Ho W.-H., Castillo P., Shinsky N., García-Verdugo J.M., Armanini M., Ryan A. (2000). Mice Lacking α-Synuclein Display Functional Deficits in the Nigrostriatal Dopamine System. Neuron.

[104] Conway K.A., Harper J.D., Lansbury P.T. (1998). Accelerated In Vitro Fibril Formation by a Mutant α-synuclein Linked to Early-Onset Parkinson Disease. Nat. Med..

[105] Greenbaum E.A., Graves C.L., Mishizen-Eberz A.J., Lupoli M.A., Lynch D.R., Englander S.W., Axelsen P.H., Giasson B.I. (2005). The E46K Mutation in α-Synuclein Increases Amyloid Fibril Formation. J. Biol. Chem..

[106] Volpicelli-Daley L.A., Luk K.C., Lee V.M.-Y. (2014). Addition of Exogenous α-Synuclein Preformed Fibrils to Primary Neuronal Cultures to Seed Recruitment of Endogenous α-Synuclein to Lewy Body and Lewy Neurite–like Aggregates. Nat. Protoc..

[107] Luk K.C., Song C., O’Brien P., Stieber A., Branch J.R., Brunden K.R., Trojanowski J.Q., Lee V.M.-Y. (2009). Exogenous α-Synuclein Fibrils Seed the Formation of Lewy Body-like Intracellular Inclusions in Cultured Cells. Proc. Natl. Acad. Sci. USA.

[108] Meier F., Abeywardana T., Dhall A., Marotta N.P., Varkey J., Langen R., Chatterjee C., Pratt M.R. (2012). Semisynthetic, Site-Specific Ubiquitin Modification of α-Synuclein Reveals Differential Effects on Aggregation. J. Am. Chem. Soc..

[109] Tanik S.A., Schultheiss C.E., Volpicelli-Daley L.A., Brunden K.R., Lee V.M.Y. (2013). Lewy Body-like α-Synuclein Aggregates Resist Degradation and Impair Macroautophagy. J. Biol. Chem..

[110] Euler U.S.V., Gaddum J.H. (1931). An Unidentified Depressor Substance in Certain Tissue Extracts. J. Physiol..

[111] Chang M.M., Leeman S.E., Niall H.D. (1971). Amino-acid Sequence of Substance P. Nat. New Biol..

[112] Nicoll R.A., Schenker C., Leeman S.E. (1980). Substance P as a Transmitter Candidate. Annu. Rev. Neurosci..

[113] Warden M.K., Young W.S. (1988). Distribution of Cells Containing mRNAs Encoding Substance P and Neurokinin B in the Rat Central Nervous System. J. Comp. Neurol..

[114] Mashaghi A., Marmalidou A., Tehrani M., Grace P.M., Pothoulakis C., Dana R. (2016). Neuropeptide Substance P and the Immune Response. Cell Mol. Life Sci..

[115] Rana T., Behl T., Sehgal A., Singh S., Sharma N., Abdeen A., Ibrahim S.F., Mani V., Iqbal M.S., Bhatia S. (2022). Exploring the Role of Neuropeptides in Depression and Anxiety. Prog. Neuro-Psychopharmacol. Biol. Psychiatry.

[116] Mauborgne A., Javoy-Agid F., Legrand J.C., Agid Y., Cesselin F. (1983). Decrease of Substance P-like Immunoreactivity in the Substantia Nigra and Pallidum of Parkinsonian Brains. Brain Res..

[117] Tenovuo O., Rinne U.K., Viljanen M.K. (1984). Substance P Immunoreactivity in the Post-mortem Parkinsonian Brain. Brain Res..

[118] Schröder J.B., Marian T., Claus I., Muhle P., Pawlowski M., Wiendl H., Suntrup-Krueger S., Meuth S.G., Dziewas R., Ruck T. (2019). Substance P Saliva Reduction Predicts Pharyngeal Dysphagia in Parkinson’s Disease. Front. Neurol..

[119] Lindefors N., Brodin E., Tossman U., Segovia J., Ungerstedt U. (1989). Tissue Levels and in Vivo Release of Tachykinins and GABA in Striatum and Substantia Nigra of Rat Brain after Unilateral Striatal Dopamine Denervation. Exp. Brain Res..

[120] Thornton E., Vink R. (2012). Treatment with a Substance P Receptor Antagonist Is Neuroprotective in the Intrastriatal 6-Hydroxydopamine Model of Early Parkinson’s Disease. PLoS ONE.

[121] Zheng Y., Zhang L., Xie J., Shi L. (2021). The Emerging Role of Neuropeptides in Parkinson’s Disease. Front. Aging Neurosci..

[122] Thornton E., Tran T.T.B., Vink R. (2010). A Substance P Mediated Pathway Contributes to 6-hydroxydopamine Induced Cell Death. Neurosci. Lett..

[123] Chu J.M.T., Chen L.W., Chan Y.S., Yung K.K.L. (2011). Neuroprotective Effects of Neurokinin Receptor One in Dopaminergic Neurons Are Mediated through Akt/PKB Cell Signaling Pathway. Neuropharmacology.

[124] Amadoro G., Pieri M., Ciotti M.T., Carunchio I., Canu N., Calissano P., Zona C., Severini C. (2007). Substance P Provides Neuroprotection in Cerebellar Granule Cells through Akt and MAPK/Erk Activation: Evidence for the Involvement of the Delayed Rectifier Potassium Current. Neuropharmacology.

[125] Chao O.Y., Wang A.-L., Nikolaus S., De Souza Silva M.A. (2015). NK3 Receptor Agonism Reinstates Temporal Order Memory in the Hemiparkinsonian Rat. Behav. Brain Res..

[126] Thornton E., Hassall M.M., Corrigan F., Vink R. (2014). The NK1 Receptor Antagonist N-acetyl-l-tryptophan Reduces Dyskinesia in a Hemi-parkinsonian Rodent Model. Park. Relat. Disord..

[127] Yang X., Zhao H., Shi H., Wang X., Zhang S., Zhang Z., Zu J., Zhang W., Shen X., Cui G. (2015). Intranigral Administration of Substance P Receptor Antagonist Attenuated Levodopa-Induced Dyskinesia in a Rat Model of Parkinson’s Disease. Exp. Neurol..

[128] Kim W.-G., Mohney R.P., Wilson B., Jeohn G.-H., Liu B., Hong J.-S. (2000). Regional Difference in Susceptibility to Lipopolysaccharide-Induced Neurotoxicity in the Rat Brain: Role of Microglia. J. Neurosci..

[129] Kojima M., Hosoda H., Date Y., Nakazato M., Matsuo H., Kangawa K. (1999). Ghrelin Is a Growth-Hormone-Releasing Acylated Peptide from Stomach. Nature.

[130] Serrenho D., Santos S.D., Carvalho A.L. (2019). The Role of Ghrelin in Regulating Synaptic Function and Plasticity of Feeding-Associated Circuits. Front. Cell. Neurosci..

[131] St-Pierre D.H., Wang L., Taché Y. (2003). Ghrelin: A Novel Player in the Gut-Brain Regulation of Growth Hormone and Energy Balance. Physiology.

[132] Frago L.M., Ebaquedano E., Eargente J., Chowen J.A. (2011). Neuroprotective Actions of Ghrelin and Growth Hormone Secretagogues. Front. Mol. Neurosci..

[133] Rees D., Johnson A.L., Lelos M., Smith G., Roberts L.D., Phelps L., Dunnett S.B., Morgan A.H., Brown R.M., Wells T. (2022). Acyl-Ghrelin Attenuates Neurochemical and Motor Deficits in the 6-OHDA Model of Parkinson’s Disease. bioRxiv.

[134] He X., Yuan W., Liu F., Feng J., Guo Y. (2021). Acylated Ghrelin is Protective Against 6-OHDA-induced Neurotoxicity by Regulating Autophagic Flux. Front. Pharmacol..

[135] Wagner J., Vulinović F., Grünewald A., Unger M.M., Möller J.C., Klein C., Michel P.P., Ries V., Oertel W.H., Alvarez-Fischer D. (2017). Acylated and Unacylated Ghrelin Confer Neuroprotection to Mesencephalic Neurons. Neuroscience.

[136] Song N., Wang W., Jia F., Du X., Xie A., He Q., Shen X., Zhang J., Rogers J., Xie J. (2017). Assessments of Plasma Ghrelin Levels in the Early Stages of Parkinson’s Disease. Mov. Disord..

[137] Andrews Z.B., Erion D., Beiler R., Liu Z.-W., Abizaid A., Zigman J., Elsworth J.D., Savitt J.M., DiMarchi R., Tschoep M. (2009). Ghrelin Promotes and Protects Nigrostriatal Dopamine Function via a UCP2-Dependent Mitochondrial Mechanism. J. Neurosci..

[138] Suda Y., Kuzumaki N., Sone T., Narita M., Tanaka K., Hamada Y., Iwasawa C., Shibasaki M., Maekawa A., Matsuo M. (2018). Down-Regulation of Ghrelin Receptors on Dopaminergic Neurons in the Substantia Nigra Contributes to Parkinson’s Disease-like Motor Dysfunction. Mol. Brain.

[139] Jiang H., Li L.-J., Wang J., Xie J.-X. (2008). Ghrelin Antagonizes MPTP-induced Neurotoxicity to the Dopaminergic Neurons in Mouse Substantia Nigra. Exp. Neurol..

[140] Wang H., Dou S., Zhu J., Shao Z., Wang C., Cheng B. (2020). Ghrelin Protects Dopaminergic Neurons against MPTP Neurotoxicity through Promoting Autophagy and Inhibiting Endoplasmic Reticulum Mediated Apoptosis. Brain Res..

[141] Moon M., Kim H.G., Hwang L., Seo J.-H., Kim S., Hwang S., Kim S., Lee D., Chung H., Oh M.S. (2009). Neuroprotective Effect of Ghrelin in the 1-Methyl-4-Phenyl-1,2,3,6-Tetrahydropyridine Mouse Model of Parkinson’s Disease by Blocking Microglial Activation. Neurotox. Res..

[142] Yu J., Xu H., Shen X., Jiang H. (2016). Ghrelin Protects MES23.5 Cells against Rotenone via Inhibiting Mitochondrial Dysfunction and Apoptosis. Neuropeptides.

[143] Liu S., Chen S., Ren J., Li B., Qin B. (2018). Ghrelin Protects Retinal Ganglion Cells against Rotenone via Inhibiting Apoptosis, Restoring Mitochondrial Function, and Activating AKT-mTOR Signaling. Neuropeptides.

[144] Bayliss J.A., Lemus M.B., Stark R., Santos V.V., Thompson A., Rees D.J., Galic S., Elsworth J.D., Kemp B.E., Davies J.S. (2016). Ghrelin-AMPK Signaling Mediates the Neuroprotective Effects of Calorie Restriction in Parkinson’s Disease. J. Neurosci..

[145] Shi L., Bian X., Qu Z., Ma Z., Zhou Y., Wang K., Jiang H., Xie J. (2013). Peptide Hormone Ghrelin Enhances Neuronal Excitability by Inhibition of Kv7/KCNQ Channels. Nat. Commun..

[146] Chang X., Ma Z., Shi L., Xie J. (2020). Effects of Ghrelin on the Electrical Activities of Substantia Nigra Dopaminergic Neurons Treated with MPP+. Neurochem. Int..

[147] Gong B., Jiao L., Du X., Li Y., Bi M., Jiao Q., Jiang H. (2020). Ghrelin Promotes Midbrain Neural Stem Cells Differentiation to Dopaminergic Neurons through Wnt/β-catenin Pathway. J. Cell. Physiol..

[148] Minalyan A., Gabrielyan L., Pietra C., Taché Y., Wang L. (2019). Multiple Beneficial Effects of Ghrelin Agonist, HM01 on Homeostasis Alterations in 6-Hydroxydopamine Model of Parkinson’s Disease in Male Rats. Front. Integr. Neurosci..

[149] Popelová A., Kákonová A., Hrubá L., Kunes J., Maletínská L., Železná B. (2018). Potential Neuroprotective and Anti-Apoptotic Properties of a Long-Lasting Stable Analog of Ghrelin: An In Vitro Study Using SH-SY5Y Cells. Physiol. Res..

[150] Morgan A.H., Rees D.J., Andrews Z.B., Davies J.S. (2018). Ghrelin Mediated Neuroprotection—A Possible Therapy for Parkinson’s Disease?. Neuropharmacology.

[151] Dong J., Song N., Xie J., Jiang H. (2009). Ghrelin Antagonized 1-Methyl-4-Phenylpyridinium (MPP+)-Induced Apoptosis in MES23.5 Cells. J. Mol. Neurosci..

[152] Liu L., Xu H., Jiang H., Wang J., Song N., Xie J. (2010). Ghrelin Prevents 1-methyl-4-phenylpyridinium Ion-induced Cytotoxicity through Antioxidation and NF-κB Modulation in MES23.5 cells. Exp. Neurol..

[153] Bayliss J.A., Andrews Z.B. (2013). Ghrelin Is Neuroprotective in Parkinson’s Disease: Molecular Mechanisms of Metabolic Neuroprotection. Ther. Adv. Endocrinol. Metab..

[154] Tatemoto K., Carlquist M., Mutt V. (1982). Neuropeptide Y—A Novel Brain Peptide with Structural Similarities to Peptide YY and Pancreatic Polypeptide. Nature.

[155] Gehlert D.R. (2004). Introduction to the Reviews on Neuropeptide Y. Neuropeptides.

[156] Tatemoto K. (1982). Neuropeptide Y: Complete Aamino Acid Sequence of the Brain Peptide. Proc. Natl. Acad. Sci. USA.

[157] Shiozaki K., Kawabe M., Karasuyama K., Kurachi T., Hayashi A., Ataka K., Iwai H., Takeno H., Hayasaka O., Kotani T. (2020). Neuropeptide Y Deficiency Induces Anxiety-like Behaviours in Zebrafish (*Danio rerio*). Sci. Rep..

[158] Pedragosa-Badia X., Stichel J., Beck-Sickinger A.G. (2013). Neuropeptide Y Receptors: How to Get Subtype Selectivity. Front. Endocrinol..

[159] Lee C.C., Miller R.J. (1998). Is There Really an NPY Y3 Receptor?. Regul. Pept..

[160] Starbäck P., Wraith A., Eriksson H., Larhammar D. (2000). Neuropeptide Y Receptor Gene y6: Multiple Deaths or Resurrections?. Biochem. Biophys. Res. Commun..

[161] dos Santos V.V., Santos D.B., Lach G., Rodrigues A.L.S., Farina M., De Lima T.C.M., Prediger R. (2013). Neuropeptide Y (NPY) Prevents Depressive-like Behavior, Spatial Memory Deficits and Oxidative Stress Following Amyloid-β (Aβ1–40) Administration in Mice. Behav. Brain Res..

[162] Malva J.O., Xapelli S., Baptista S., Valero J., Agasse F., Ferreira R., Silva A.P. (2012). Multifaces of Neuropeptide Y in the Brain—Neuroprotection, Neurogenesis and Neuroinflammation. Neuropeptides.

[163] Stoddard S.L., Tyce G.M., Ahlskog J., Zinsmeister A.R., Nelson D.K., Carmichael S.W. (1991). Decreased Levels of [Met]Enkephalin, Neuropeptide Y, Substance P, and Vasoactive Intestinal Peptide in Parkinsonian Adrenal Medulla. Exp. Neurol..

[164] Martignoni E., Blandini F., Petraglia F., Pacchetti C., Bono G., Nappi G. (1992). Cerebrospinal Fluid Norepinephrine, 3-Methoxy-4-Hydroxyphenylglycol and Neuropeptide Y Levels in Parkinson’s Disease, Multiple System Atrophy and Dementia of the Alzheimer Type. J. Neural Transm..

[165] Cannizzaro C., Tel B.C., Rose S., Zeng B.-Y., Jenner P. (2003). Increased Neuropeptide Y mRNA Expression in Striatum in Parkinson’s Disease. Mol. Brain Res..

[166] Goto S., Kawarai T., Morigaki R., Okita S., Koizumi H., Nagahiro S., Munoz E.L., Lee L.V., Kaji R. (2013). Defects in the Striatal Neuropeptide Y System in X-Linked Dystonia-Parkinsonism. Brain.

[167] Svenningsson P., Pålhagen S., Mathé A.A. (2017). Neuropeptide Y and Calcitonin Gene-Related Peptide in Cerebrospinal Fluid in Parkinson’s Disease with Comorbid Depression versus Patients with Major Depressive Disorder. Front. Psychiatry.

[168] Obuchowicz E., Antkiewicz-Michaluk L., Romańska I., Herman Z.S. (2003). Increased Striatal Neuropeptide Y Immunoreactivity and Its Modulation by Deprenyl, Clonidine and L-dopa in MPTP-treated Mice. J. Neural Transm..

[169] Decressac M., Pain S., Chabeauti P.-Y., Frangeul L., Thiriet N., Herzog H., Vergote J., Chalon S., Jaber M., Gaillard A. (2012). Neuroprotection by Neuropeptide Y in Cell and Animal Models of Parkinson’s Disease. Neurobiol. Aging.

[170] Pain S., Vergote J., Gulhan Z., Bodard S., Chalon S., Gaillard A. (2019). Inflammatory Process in Parkinson Disease: Neuroprotection by Neuropeptide Y. Fundam. Clin. Pharmacol..

[171] Ferreira R., Xapelli S., Santos T., Silva A.P., Cristóvão A., Cortes L., Malva J.O. (2010). Neuropeptide Y Modulation of Interleukin-1β (IL-1β)-induced Nitric Oxide Production in Microglia. J. Biol. Chem..

[172] Lee D.Y., Hong S.H., Kim B., Lee D.S., Yu K., Lee K.-S. (2018). Neuropeptide Y Mitigates ER Stress–induced Neuronal Cell Death by Activating the PI3K–XBP1 Pathway. Eur. J. Cell Biol..

[173] Sendtner M., Holtmann B., Kolbeck R., Thoenen H., Barde Y. (1992). Brain-Derived Neurotrophic Factor Prevents the Death of Motoneurons in Newborn Rats after Nerve Section. Nature.

[174] Fumagalli F., Racagni G., Riva M.A. (2006). Shedding Light into the Role of BDNF in the Pharmacotherapy of Parkinson’s Disease. Pharm. J..

[175] Carraway R., Leeman S.E. (1973). The Isolation of a New Hypotensive Peptide, Neurotensin, from Bovine Hypothalami. J. Biol. Chem..

[176] Russjan E., Kaczyńska K. (2019). Beneficial Effects of Neurotensin in Murine Model of Hapten-Induced Asthma. Int. J. Mol. Sci..

[177] Carraway R., Leeman S.E. (1975). The Amino Acid Sequence of a Hypothalamic Peptide, Neurotensin. J. Biol. Chem..

[178] St.-Gelais F., Jomphe C., Trudeau L.É.. (2006). The Role of Neurotensin in Central Nervous System Pathophysiology: What Is the Evidence?. J. Psychiatry Neurosci..

[179] Wu Z., Martinez-Fong D., Trédaniel J., Forgez P. (2013). Neurotensin and Its High Affinity Receptor 1 as a Potential Pharmacological Target in Cancer Therapy. Front. Endocrinol..

[180] Mazella J., Béraud-Dufour S., Devader C., Massa F., Coppola T. (2012). Neurotensin and Its Receptors in the Control of Glucose Homeostasis. Front. Endocrinol..

[181] Jomphe C., Lemelin P.-L., Okano H., Kobayashi K., Trudeau L.-E. (2006). Bidirectional Regulation of Dopamine D2 and Neurotensin NTS1 Receptors in Dopamine Neurons. Eur. J. Neurosci..

[182] Fernandez A., de Ceballos M.L., Rose S., Jenner P., Marsden C.D. (1996). Alterations in Peptide Levels in Parkinson’s Disease and Incidental Lewy Body Disease. Brain.

[183] Fernandez A., Jenner P., Marsden C.D., de Ceballos M.L. (1995). Characterization of Neurotensin-like Immunoreactivity in Human Basal Ganglia: Increased Neurotensin Levels in Substantia Nigra in Parkinson’s Disease. Peptides.

[184] Schimpff R.-M., Avard C., Fénelon G., Lhiaubet A.-M., Tennezé L., Vidailhet M., Rostène W. (2001). Increased Plasma Neurotensin Concentrations in Patients with Parkinson’s Disease. J. Neurol. Neurosurg. Psychiatry.

[185] Yamada M., Richelson E. (1995). Heterogeneity of Melanized Neurons Expressing Neurotensin Receptor Messenger RNA in the Substantia Nigra and the Nucleus Paranigralis of Control and Parkinson’s Disease Brain. Neuroscience.

[186] Uhl G.R., Whitehouse P.J., Price D.L., Tourtelotte W.W., Kuhar M.J. (1984). Parkinson’s Disease: Depletion of Substantia Nigra Neurotensin Receptors. Brain Res..

[187] Fernandez A., de Ceballos M.L., Jenner P., Marsden C.D. (1994). Neurotensin, Substance P, Delta and Mu Opioid Receptors Are Decreased in Basal Ganglia of Parkinson’s Disease Patients. Neuroscience.

[188] Chinaglia G., Probst A., Palacios J. (1990). Neurotensin Receptors in Parkinson’s Disease and Progressive Supranuclear Palsy: An Autoradiographic Study in Basal Ganglia. Neuroscience.

[189] Rivest R., St-Pierre S., Jolicoeur F.B. (1991). Structure-Activity Studies of Neurotensin on Muscular Rigidity and Tremors Induced by 6-Hydroxydopamine Lesions in the Posterolateral Hypothalamus of the Rat. Neuropharmacology.

[190] Antonelli T., Fuxe K., Tomasini M.C., Mazzoni E., Agnati L.F., Tanganelli S., Ferraro L. (2007). Neurotensin Receptor Mechanisms and Its Modulation of Glutamate Transmission in the Brain: Relevance for Neurodegenerative Diseases and Their Treatment. Prog. Neurobiol..

[191] Gilmartin M.R., Ferrara N.C. (2021). Pituitary Adenylate Cyclase-Activating Polypeptide in Learning and Memory. Front. Cell. Neurosci..

[192] Miyata A., Arimura A., Dahl R.R., Minamino N., Uehara A., Jiang L., Culler M.D., Coy D.H. (1989). Isolation of a Novel 38 Residue-Hypothalamic Polypeptide Which Stimulates Adenylate Cyclase in Pituitary Cells. Biochem. Biophys. Res. Commun..

[193] Liao C., De Molliens M.P., Schneebeli S.T., Brewer M., Song G., Chatenet D., Braas K.M., May V., Li J. (2019). Targeting the PAC1 Receptor for Neurological and Metabolic Disorders. Curr. Top. Med. Chem..

[194] Shioda S., Takenoya F., Wada N., Hirabayashi T., Seki T., Nakamachi T. (2016). Pleiotropic and Retinoprotective Functions of PACAP. Anat. Sci. Int..

[195] Lamine-Ajili A., Fahmy A.M., Létourneau M., Chatenet D., Labonté P., Vaudry D., Fournier A. (2016). Effect of the Pituitary Adenylate Cyclase-Activating Polypeptide on the Autophagic Activation Observed in in Vitro and in Vivo Models of Parkinson’s Disease. Biochim. Biophys. Acta (BBA)—Mol. Basis Dis..

[196] Hirabayashi T., Shibato J., Kimura A., Yamashita M., Takenoya F., Shioda S. (2022). Potential Therapeutic Role of Pituitary Adenylate Cyclase-Activating Polypeptide for Dry Eye Disease. Int. J. Mol. Sci..

[197] Saklani P., Khan H., Gupta S., Kaur A., Singh T.G. (2022). Neuropeptides: Potential Neuroprotective Agents in Ischemic Injury. Life Sci..

[198] Hannibal J. (2002). Pituitary Adenylate Cyclase-Activating Peptide in the Rat Central Nervous System: An Immunohistochemical and in Situ Hybridization Study. J. Comp. Neurol..

[199] Feher M., Gaszner B., Tamas A., Gil-Martinez A.L., Fernandez-Villalba E., Herrero M.T., Reglodi D. (2018). Alteration of the PAC1 Receptor Expression in the Basal Ganglia of MPTP-Induced Parkinsonian Macaque Monkeys. Neurotox. Res..

[200] Reglodi D., Renaud J., Tamas A., Tizabi Y., Socías S.B., Del-Bel E., Raisman-Vozari R. (2017). Novel Tactics for Neuroprotection in Parkinson’s Disease: Role of Antibiotics, Polyphenols and Neuropeptides. Prog. Neurobiol..

[201] Reglodi D., Kiss P., Lubics A., Tamas A. (2011). Review on the Protective Effects of PACAP in Models of Neurodegenerative Diseases In Vitro and In Vivo. Curr. Pharm. Des..

[202] Reglodi D., Lubics A., Tamás A., Szalontay L., Lengvári I. (2004). Pituitary Adenylate Cyclase Activating Polypeptide Protects Dopaminergic Neurons and Improves Behavioral Deficits in a Rat Model of Parkinson’s Disease. Behav. Brain Res..

[203] Reglődi D., Tamás A., Lubics A., Szalontay L., Lengvári I. (2004). Morphological and Functional Effects of PACAP in 6-Hydroxydopamine-Induced Lesion of the Substantia Nigra in Rats. Regul. Pept..

[204] Deguil J., Chavant F., Lafay-Chebassier C., Pérault-Pochat M.-C., Fauconneau B., Pain S. (2010). Neuroprotective Effect of PACAP on Translational Control Alteration and Cognitive Decline in MPTP Parkinsonian Mice. Neurotox. Res..

[205] Wang G., Qi C., Fan G.-H., Zhou H.-Y., Chen S.-D. (2005). PACAP Protects Neuronal Differentiated PC12 Cells against the Neurotoxicity Induced by a Mitochondrial Complex I Inhibitor, Rotenone. FEBS Lett..

[206] Brown D., Tamas A., Reglodi D., Tizabi Y. (2013). PACAP Protects Against Salsolinol-Induced Toxicity in Dopaminergic SH-SY5Y Cells: Implication for Parkinson’s Disease. J. Mol. Neurosci..

[207] Shivers K.-Y., Nikolopoulou A., Machlovi S.I., Vallabhajosula S., Figueiredo-Pereira M.E. (2014). PACAP27 Prevents Parkinson-like Neuronal Loss and Motor Deficits but Not Microglia Activation Induced by Prostaglandin J2. Biochim. Biophys. Acta (BBA)—Mol. Basis Dis..

[208] Brown D., Tamás A., Reglodi D., Tizabi Y. (2014). PACAP Protects Against Inflammatory-Mediated Toxicity in Dopaminergic SH-SY5Y Cells: Implication for Parkinson’s Disease. Neurotox. Res..

[209] Wang G., Pan J., Tan Y.-Y., Sun X.-K., Zhang Y.-F., Zhou H.-Y., Ren R.-J., Wang X.-J., Chen S.-D. (2008). Neuroprotective Effects of PACAP27 in Mice Model of Parkinson’s Disease Involved in the Modulation of KATP Subunits and D2 Receptors in the Striatum. Neuropeptides.

[210] Yang S., Yang J., Yang Z., Chen P., Fraser A., Zhang W., Pang H., Gao X., Wilson B., Hong J.-S. (2006). Pituitary Adenylate Cyclase-Activating Polypeptide (PACAP) 38 and PACAP4–6 Are Neuroprotective through Inhibition of NADPH Oxidase: Potent Regulators of Microglia-Mediated Oxidative Stress. J. Pharmacol. Exp. Ther..

[211] Said S.I., Mutt V. (1970). Polypeptide with Broad Biological Activity: Isolation from Small Intestine. Science.

[212] Korkmaz O.T., Tunçel N. (2019). Advantages of Vasoactive Intestinal Peptide for the Future Treatment of Parkinson’s Disease. Curr. Pharm. Des..

[213] Delgado M., Ganea D. (2003). Neuroprotective Effect of Vasoactive Intestinal Peptide (VIP) In a Mouse Model of Parkinson’s Disease by Blocking Microglial Activation. FASEB J..

[214] Broome S.T., Musumeci G., Castorina A. (2022). PACAP and VIP Mitigate Rotenone-Induced Inflammation in BV-2 Microglial Cells. J. Mol. Neurosci..

[215] Oh I.S., Shimizu H., Satoh T., Okada S., Adachi S., Inoue K., Eguchi H., Yamamoto M., Imaki T., Hashimoto K. (2006). Identification of Nesfatin-1 as a Satiety Molecule in the Hypothalamus. Nature.

[216] Rupp S.K., Wölk E., Stengel A. (2021). Nesfatin-1 Receptor: Distribution, Signaling and Increasing Evidence for a G Protein-Coupled Receptor—A Systematic Review. Front. Endocrinol..

[217] Goebel-Stengel M., Wang L. (2013). Central and Peripheral Expression and Distribution of NUCB2/nesfatin-1. Curr. Pharm. Des..

[218] Shimizu H., Mori M. (2013). Nesfatin-1: Its Role in the Diagnosis and Treatment of Obesity and Some Psychiatric Disorders. Calcium-Bind. Proteins RAGE.

[219] Chen H., Li X., Ma H., Zheng W., Shen X. (2021). Reduction in Nesfatin-1 Levels in the Cerebrospinal Fluid and Increased Nigrostriatal Degeneration Following Ventricular Administration of Anti-nesfatin-1 Antibody in Mice. Front. Neurosci..

[220] Price T.O., Samson W.K., Niehoff M.L., Banks W.A. (2007). Permeability of the Blood–Brain Barrier to a Novel Satiety Molecule Nesfatin-1. Peptides.

[221] Emir G.K., Ünal Y., Yılmaz N., Tosun K., Kutlu G. (2019). The Association of Low Levels of Nesfatin-1 and Glucagon-like Peptide-1 with Oxidative Stress in Parkinson’s Disease. Neurol. Sci..

[222] Tang C.-H., Fu X.-J., Xu X.-L., Wei X.-J., Pan H.-S. (2012). The Anti-inflammatory and Anti-apoptotic Effects of Nesfatin-1 in the Traumatic Rat Brain. Peptides.

[223] Özsavcí D., Erşahin M., Şener A., Özakpinar Ö.B., Toklu H.Z., Akakín D., Şener G., Yeğen B. (2011). The Novel Function of Nesfatin-1 as an Anti-inflammatory and Antiapoptotic Peptide in Subarachnoid Hemorrhage–Induced Oxidative Brain Damage in Rats. Neurosurgery.

[224] Tan Z., Xu H., Shen X., Jiang H. (2015). Nesfatin-1 Antagonized Rotenone-Induced Neurotoxicity in MES23.5 Dopaminergic Cells. Peptides.

[225] Erfani S., Moghimi A., Aboutaleb N., Khaksari M. (2019). Protective Effects of Nesfatin-1 Peptide on Cerebral Ischemia Reperfusion Injury via Inhibition of Neuronal Cell Death and Enhancement of Antioxidant Defenses. Metab. Brain Dis..

[226] Shen X.-L., Song N., Du X.-X., Li Y., Xie J.-X., Jiang H. (2017). Nesfatin-1 Protects Dopaminergic Neurons against MPP+/MPTP-Induced Neurotoxicity through the C-Raf–ERK1/2-Dependent Anti-Apoptotic Pathway. Sci. Rep..

[227] Brazeau P., Vale W., Burgus R., Guillemin R. (1974). Isolation of Somatostatin (a Somatotropin Release Inhibiting Factor) of Ovine Hypothalamic Origin. Can. J. Biochem..

[228] Patel Y.C. (1999). Somatostatin and Its Receptor Family. Front. Neuroendocr..

[229] Kumar U., Grant M., Rehfeld J., Bundgaard J. (2010). Somatostatin and Somatostatin Receptors. Results and Problems in Cell Differentiation.

[230] O’Toole T.J., Sharma S. (2019). Physiology, Somatostatin. StatPearls.

[231] Weckbecker G., Lewis I., Albert R., Schmid H.A., Hoyer D., Bruns C. (2003). Opportunities in Somatostatin Research: Biological, Chemical and Therapeutic Aspects. Nat. Rev. Drug Discov..

[232] Reubi J.C. (1992). Somatostatin Receptors in the Gastrointestinal Tract in Health and Disease. Yale J. Biol. Med..

[233] Reichlin S. (1983). Somatostatin (Second of Two Parts). N. Engl. J. Med..

[234] Reichlin S. (1983). Somatostatin (First of Two Parts). N. Engl. J. Med..

[235] Behl T., Rachamalla M., Najda A., Sehgal A., Singh S., Sharma N., Bhatia S., Al-Harrasi A., Chigurupati S., Vargas-De-La-Cruz C. (2021). Applications of Adductomics in Chemically Induced Adverse Outcomes and Major Emphasis on DNA Adductomics: A Pathbreaking Tool in Biomedical Research. Int. J. Mol. Sci..

